# Sc/Mg Co‐Doping in Na_3_Zr_2_Si_2_PO_12_ Solid‐State Electrolytes Enables Outstanding Performance of Sodium Metal Batteries

**DOI:** 10.1002/advs.202515463

**Published:** 2025-09-24

**Authors:** Xin Wang, Jiayang Li, Zewei Hu, Xinghan Li, Liyang Liu, Jiazhao Wang, Jung Ho Kim, Weijie Li, Wei Kong Pang, Bernt Johannessen

**Affiliations:** ^1^ Faculty of Engineering and Information Sciences University of Wollongong Wollongong NSW 2500 Australia; ^2^ Australian Synchrotron ANSTO Clayton VIC 3168 Australia; ^3^ Powder Metallurgy Research Institute Central South University Changsha 410083 China; ^4^ Wenzhou University Technology Innovation Institute for Carbon Neutralization Wenzhou 325035 China

**Keywords:** interfacial chemistry, ionic conductivities, NASICON solid‐state electrolytes, sodium metal batteries

## Abstract

All solid‐state sodium metal batteries offer a transformative opportunity for more sustainable energy storage, with the potential to significantly improve both energy density and safety compared to conventional sodium‐ion batteries. However, their practical application is hindered by challenges such as dendrite formation and limited ion conductivity. In this study, a novel strategy is proposed in which Sc^3+^ and Mg^2+^ dopants are directly introduced into the Na_3_Zr_2_Si_2_PO_12_ solid‐state electrolyte (NZSP SSE) to optimize composition and regulate interfacial chemistry. The resulting co‐doped electrolyte, Na_3.7_Zr_1.45_Sc_0.4_Mg_0.15_Si_2_PO_12_ (NSZSP‐0.15Mg), exhibits a significantly enhanced ionic conductivity of 1.3 mS cm^−1^ at room temperature and improved interfacial compatibility. Moreover, symmetric Na//NSZSP‐0.15Mg//Na cells demonstrate stable Na stripping/plating for over 400 h (0.5 mA cm^−2^, 0.5 mAh cm^−2^), attributed to the formation of a dynamically stable ScPO_4_/Mg_3_(PO_4_)_2_‐rich interphase layer at the Na metal/SSE interface. Furthermore, Na//NSZSP‐0.15Mg//Na_3_V_2_(PO_4_)_3_ full cell batteries exhibit excellent rate capability and long‐term cycling stability, maintaining 75 mAh g^−1^ over 3000 cycles at 2 C at room temperature. This work presents a robust approach for enabling practical solid‐state sodium metal batteries with high conductivity, enhanced interfacial stability, high energy density, and long‐term cyclability, advancing the development of next‐generation SSEs for high‐performance sodium metal batteries.

## Graphical abstract

This study explores an aliovalent cation co‐doping strategy involving Sc^3+^/Mg^2+^ substitution in Na_3_Zr_2_Si_2_PO_12_ solid‐state electrolyte for high‐performance sodium metal batteries. The optimal Na_3.7_Zr_1.45_Sc_0.4_Mg_0.15_Si_2_PO_12_ SSE significantly enhances the ionic conductivity and regulates the interfacial chemistry, resulting in outstanding rate capability and reversibility, which shows promising application potential in SSSMBs.

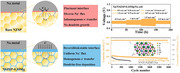

## 1 Introduction

All‐solid‐state sodium metal batteries (ASSSMBs) are poised to revolutionize grid‐scale stationary energy storage, owing to their potential for higher volumetric and gravimetric energy densities along with improved safety compared to conventional liquid electrolyte‐based systems.^[^ ^1^ ^]^ Na metal, the commonly used anode material in these battery systems, is geographically widespread and abundantly available, further enhancing the appeal of ASSSMBs for large‐scale applications.^[^ ^2^ ^]^ However, the practical deployment of Na metal anodes remains challenging due to issues such as nonuniform Na stripping/plating and dendrite formation, which severely impair interfacial kinetics.^[^ ^3^ ^]^ Solid‐state electrolytes (SSEs) with sufficient ionic conductivity at room temperature, excellent electrochemical and chemical stability with electrodes, and mechanical deformability to maintain intimate electrode‐electrolyte contact are essential for realizing high‐performance ASSSMBs.^[^ ^4^ ^]^ Unfortunately, the inherent sluggish ionic conductivity of many SSEs and poor interfacial contact with electrodes limit suitable SSE options and contribute to dendrite penetration, respectively. Therefore, the development of novel SSEs must address both intrinsic properties and interfacial compatibility with Na metal to advance next‐generation ASSSMBs.^[^ ^5^ ^]^

SSEs for ASSSMBs can generally be categorized into three main classes: inorganic SSEs, including sulfides (Na_3_SbS_4_, Na_11_Sn_2_PS_12_), oxides (Na‐β″‐Al_2_O_3_, sodium superionic conductor (NASICON) type), and halides (NaNbCl_6_, Na_2_ZrCl_6_), polymer SSEs, and inorganic‐polymer hybrid SSEs.^[^ ^6^ ^]^ Among these, NASICON‐structured compounds with the general formula Na_1+x_Zr_2_Si_x_P_3‐x_O_12_ (0≤x≤3, referred to as NZSP) have attracted significant attention due to their moderate room‐temperature ionic conductivity (on the order of mS cm^−1^), excellent environmental and chemical stability (particularly toward Na metal), robust mechanical properties, and compositional tunability.^[^ ^2^, ^7^ ^]^ However, achieving ionic conductivities in NZSP that rival those of commercial organic liquid electrolytes remains a significant challenge, particularly under ambient conditions. In addition, NZSP materials often require high‐temperature sintering and rigid ceramic processing, which can lead to surface roughness and mechanical rigidity, thereby hindering intimate interfacial contact with electrodes. These factors collectively present barriers to large‐scale processability and practical implementation.

To effectively enhance the intrinsic ionic conductivity of NZSP SSEs and to tune interfacial chemistry at the electrode‐electrolyte interface, extensive research efforts have been made in recent decades. Strategic selection of dopant elements, either via aliovalent doping or isovalent substitution at specific lattice sites within the NZSP structure, is widely recognized as one of the most effective approaches to enhance bulk ionic conductivity.^[^ ^8^ ^]^ For example, Ma et al. proposed that Sc^3+^ is a promising substitution ion for NZSP, as its ionic radius (r = 0.745 Å) closely matches that of Zr^4+^ (r = 0.72 Å); their Sc‐doped Na_3.4_Sc_0.4_Zr_1.6_(SiO_4_)_2_(PO_4_) SSE exhibited an optimum total ionic conductivity at room temperature.^[^ ^9^ ^]^ Similarly, Mg^2+^ has emerged as an effective dopant; Wang et al. synthesized this unique Na_3.25_Zr_1.75_Mg_0.25_Si_2_PO_12_ SSE and delivered an adequate ionic conductivity.^[^ ^10^ ^]^ He et al. investigated the ionic conductivity and interfacial performance of NZSP SSEs by using Ni^2+^, Mn^3+^, Nb^5+^, Mo^6+^ substituting Zr^4+^ sites.^[^ ^11^ ^]^ Their results revealed that low‐valence dopants, such as Ni^2+^ and Mn^3+^, can expand lattice bottlenecks and reduce grain boundary resistance, facilitating Na^+^ migration. In contrast high‐valence dopants, such as Nb^5+^ and Mo^6+^ tend to lower migration barriers at grain boundaries. However, single‐doped strategies suffer from inherent limitations like excessive substitution or Na^+^ mobile defects, thereby leading to trade‐offs between ionic conductivity, structural stability, and interfacial compatibility with Na metal, making it challenging to achieve a balanced optimization of all performance parameters. These drawbacks highlight the need for co‐doping approaches that can integrate the distinct advantages of different dopants while mitigating their individual shortcomings.

In parallel, several complementary strategies have been developed to improve interfacial contact and reduce interfacial resistance, including surface environment modification,^[^ ^12^ ^]^ ionic liquid phase wetting regulation,^[^ ^13^ ^]^ and the introduction of interlayers or interphases via coating techniques.^[^ ^14^ ^]^ Nevertheless, these external modifications typically require complex fabrication processes and may introduce additional compatibility issues if not carefully optimized. SSEs have yet to be identified that fully satisfy the mechanical, chemical, electrochemical, and economic requirements necessary to realize high‐performance ASSSMBs. Clearly, further advances in SSE design and engineering, particularly for NZSP‐based systems, are urgently needed to enable their practical implementation in ASSSMBs.

Herein, we systematically investigate an aliovalent cation co‐doping strategy involving Sc^3+^/Mg^2+^ substitution in NZSP SSE to enhance ionic conductivity and interfacial properties, aiming to advance room‐temperature ASSSMBs with long‐term cycling stability and excellent rate performance. Sc^3+^/Mg^2+^ substitutions at the Zr^4+^ site provide distinct but complementary effects on the structure and defect chemistry. Sc^3+^ contributes to increased Na^+^ vacancy concentration, enhancing charge carrier density, while Mg^2+^ mitigates local lattice distortion and broadens Na^+^ migration pathways. The combination of Sc‐induced vacancies and Mg‐induced lattice relaxation enables a more optimized and continuous 3D Na^+^ conduction network, thereby contributing to the modified NZSP structure. By optimizing the Mg doping ratio, the resulting composition Na_3.7_Zr_1.45_Sc_0.4_Mg_0.15_Si_2_PO_12_ (NSZSP‐0.15Mg) achieves an ionic conductivity of 1.3 mS cm^−1^ at room temperature, which is over 13 times higher than that of undoped NZSP. The co‐doped Sc^3+^ and Mg^2+^ species play a dual role, not only enhancing bulk ion transport but also improving compatibility with Na metal by promoting the formation of dynamically stable ScPO_4_/Mg_3_(PO_4_)_2_‐rich interphase. This interface ensures efficient Na^+^ transport and suppresses dendrite growth, enabling dendrite‐free Na stripping/plating at the electrodes‐electrolyte interface. As a result, a symmetric Na//NSZSP‐0.15Mg//Na cell demonstrates stable Na metal deposition/dissolution for over 400 h at 0.5 mA cm^−2^, achieving a cumulative capacity of 400 mAh cm^−2^. Furthermore, in a room‐temperature solid‐state full cell configuration with a Na_3_V_2_(PO_4_)_3_ cathode, the NSZSP‐0.15Mg SSE delivers a high specific capacity of 75 mAh g^−1^ over 3000 cycles with 64% retention at a high rate of 2 C, highlighting its excellent rate capability and long‐term stability. This work demonstrates a promising and flexible strategy for enabling practical, high‐performance ASSSMBs through co‐doping design and interfacial engineering.

## 2 Results and Discussion

**Figure 1:**
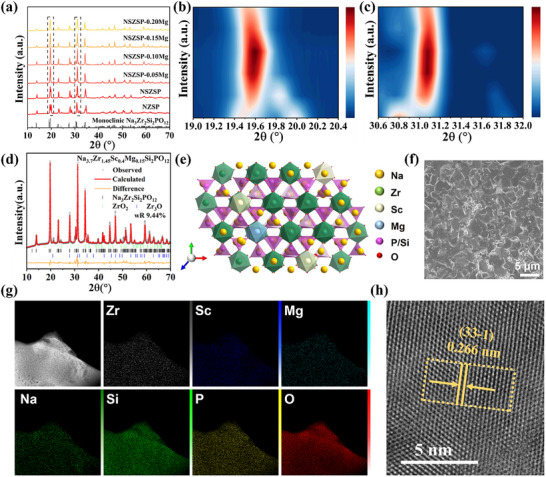
a) XRD patterns of Na_3.4+2x_Zr_1.6‐x_Sc_0.4_Mg_x_Si_2_PO_12_ (x = 0.05, 0.10, 0.15, 0.20) samples and the Contour Maps of XRD patterns in the selected 2θ range of b) 19–20.4° and c) 30.3–35.0°. d) Rietveld refinement result of the XRD pattern of NSZSP‐0.15Mg. e) The crystal structure of NSZSP‐xMg SSEs. f) Top‐view surface SEM image of NSZSP‐0.15Mg SSE. g) TEM and EDS mapping images of NSZSP‐0.15Mg SSE. h) HRTEM of NSZSP‐0.15Mg SSE.

Sc^3+^/Mg^2+^ co‐doped NZSP materials with a chemical formula of Na_3.4+2x_Zr_1.6‐x_Sc_0.4_Mg_x_Si_2_PO_12_ (defined as NSZSP‐xMg, x = 0.05, 0.10, 0.15, 0.20) were successfully synthesized via a general two‐step solid‐state reaction method.^[^ ^15^ ^]^ Figure S1 (Supporting Information) shows the obtained NZSP SSEs pellets with a diameter of 13 mm and a thickness of 0.9 mm. Detailed synthesis processes are given in the experimental section. Notably, the molar ratio of Sc is fixed at a doping level of 0.4, as it has been reported that the introduction of Sc^3+^ at the Zr‐site introduces Na^+^ vacancies for charge compensation and promotes structural symmetry, which increases mobile ion concentration.^[^ ^9^ ^]^ Simultaneously, the NASICON structure displays an optimum ionic conductivity when the effective mean ionic radius is as close to Zr^4+^ (r = 0.72 Å) as possible.^[^ ^16^ ^]^ The substituted ions at the Zr‐site should have the same or similar size for a more rational structure. Hence, a co‐doping of Sc^3+^ (r = 0.745 Å) and Mg^2+^ (r = 0.72 Å) ions is selected, as the introduction of Mg^2+^ effectively weakens the charge compensation effect and helps relieve local lattice strain, thereby slightly expanding Na^+^ migration bottlenecks. The Powder X‐ray diffraction (XRD) measurements were first employed to investigate the phase formation, structural variations, and transitions of the undoped and the doped NZSP SSEs. As depicted in **Figure** **1**a, the samples are indexed to the standard monoclinic Na_3_Zr_2_Si_2_PO_12_ phase associated with space group C2/c, with the main diffraction peaks near 2θ≈19°, 24°, 28°, 32°, 35°. A slight displacement of the XRD patterns for the NSZSP‐xMg samples relative to the standard reference can be attributed to minor instrumental calibration in NZSP sample and primarily a lattice distortion caused by the substitution of Sc^3+^ and Mg^2+^ for Zr^4+^ in the NSZSP‐xMg framework. Specifically, as the Mg content increases, the two split peaks near 19° progressively converge into a single dominant peak, with the decrease in content of the second impure phase, which represents enhanced phase stability, increased structural symmetry, and reduced lattice distortion.^[^ ^17^ ^]^ Concurrently, the reduction in peak intensity implies grain refinement, where smaller particle sizes contribute to enhanced ionic transport. Additional diffraction peaks near 2θ ≈ 21.5°, 28.2°, 31.5° are attributed to the formation of ZrO_2_ and Zr_3_O phases, which arise from the inevitable volatilization of Na and P during the high‐temperature sintering process.^[^ ^18^ ^]^ The presence of this second phase ZrO_2_ in the continuous NASICON framework is electrically insulating and ionically inactive, which will interrupt the 3D Na^+^ conduction network, leading to lower total ionic conductivity. A pronounced decrease of impurity ZrO_2_ peaks intensity after substituting Mg is observed, which might result from the replacement Zr with Mg, hindering the Zr forming ZrO_2_ with excess oxygen that existed after the release of Si/P. This is beneficial for improving the phase purity of the synthesized samples. Moreover, the absence of Sc^3+^/Mg^2+^‐containing impurity phases in the diffraction patterns confirms the successful doping of Sc^3+^ and Mg^2+^ ions into the NZSP lattice. This tendency can be more obvious when conducting XRD measurements at the selected 2θ ranges of 19‐20.4° and 30.6‐32°, with the corresponding contour map plots presented in Figure 1b,c, respectively. With increasing Sc^3+^/Mg^2+^ doping levels, pronounced peak shifts are observed across multiple diffraction reflections. Figure 1d depicts the Rietveld refined XRD patterns of the NSZSP‐0.15Mg SSE to investigate the crystallographic structure change. The refined XRD patterns of the undoped NZSP, Sc^3+^ doped NSZSP, and other Sc^3+^/Mg^2+^ doped NZSP SSEs are also delineated in Figure S2 (Supporting Information). Correspondingly, Table S1 and Figure S3 (Supporting Information) summarize the variations in undoped and doped NZSP unit cell lattice parameters as revealed by XRD Rietveld refinement. Owing to the mild structural modulation induced by Sc^3+^ incorporation, no significant variation in lattice parameters was observed when Sc^3+^ was solely introduced into the NZSP framework. Upon the incorporation of Mg^2+^ ion into the NSZSP structure, the unit cell parameters *a*‐, *b*‐ and *c*‐axis exhibit a systematic increase, indicating lattice modification induced by Mg^2+^ doping. Specifically, Mg^2+^ doping in Zr^4+^ sites creates a net negative charge in the lattice, which, in an ideal stoichiometric scenario, is compensated by adding extra Na^+^ to the structure, thereby avoiding the creation of Na^+^ vacancies. However, during high‐temperature processing, partial Na loss through volatilization or redistribution can occur, leading to the formation of additional Na^+^ vacancies. In parallel, subtle changes in the oxidation state of framework elements may also contribute to charge compensation. These compensations can distort the lattice and cause anisotropic or overall expansion of unit cell parameters. Concurrently, Zr‐O bonds are generally more covalent and directional compared to the more ionic and flexible Mg‐O bonds, which contribute to a more flexible structure and introduce greater distortion or tilting of the Sc/MgO_6_ octahedra and adjacent SiO_4_/PO_4_ tetrahedra, thereby causing the relaxation of bond angles and lengths.^[^ ^19^ ^]^ However, when the Mg content is raised to 0.20, a slight contraction in lattice parameters is observed, suggesting that excessive doping may lead to over‐lattice distortion, Mg‐related secondary phase formation, or the solubility limit being reached. Sc^3+^/Mg^2+^ doping appears to collectively enlarge the critical bottlenecks associated with Na ion migration by reducing the local electrostatic field strength and introducing lattice relaxation.^[^ ^20^ ^]^ The NSZSP‐0.15 SSE with a moderate Mg^2+^ incorporation contributes to enhanced structural stability and lattice openness, which facilitates the construction of ion migration pathways and thus improves the ionic conductivity. Figure 1e demonstrates the corresponding crystal structure of NSZSP‐xMg samples, co‐doped Sc^3+^ and Mg^2+^ ions partially substitute for Zr^4+^ ions at the (Zr, Sc/Mg)O_6_ octahedral sites. Enhanced Na mobility in the NZSP structure is attributed to its higher symmetry and broader diffusion pathways. A scanning electron microscope (SEM) and a transmission electron microscope (TEM) outfitted with an energy‐dispersive spectrometer (EDS) method are provided to investigate the morphology and microstructure of NSZSP‐0.15Mg ceramic particles. Figure 1f shows the top‐view surface SEM image of the NSZSP‐0.15Mg SSE, revealing a denser microstructure and almost no defects on the SSE surface, which will further suppress Na permeation or dendrites and enhance the uniform Na/NSZSP‐0.15Mg SSE interface. Top‐view surface SEM images of the other synthesized NZSP SSEs are depicted in Figure S4 (Supporting Information), various degrees of defects, including grain boundary voids, can be observed. Moreover, the cross‐section view SEM images of SSEs are also displayed in Figure S5 (Supporting Information), which demonstrate a consistent tendency with the top‐view surface SEM results. The dark‐field STEM image and corresponding EDS mapping of the NSZSP‐0.15Mg SSE particles, shown in Figure 1g, reveal a homogeneous distribution of Na, Zr, Sc, Mg, Si, P, and O without forming a doped elements‐related secondary phase, indicating the successful introduction of Sc^3+^/Mg^2+^ co‐doping into the NZSP main phase. High‐resolution transmission electron microscopy (HRTEM) image in Figure 1h displays an interplanar distance of 0.266 nm. Well‐defined lattice fringes associated with the (33‐1) planes and their corresponding diffraction spots are evident, confirming the presence of a NASICON structure. The selected area electron diffraction (SAED) pattern (Figure S6, Supporting Information) displays well‐defined and intense diffraction rings, which are indexed to the (740), (‐7‐40), (33‐1), (‐3‐31), (411), (‐4‐1‐1), (1‐22), and (‐12‐2) crystallographic planes of the primary NASICON phase. This observation is consistent with the results obtained from XRD and HRTEM, further confirming the phase identification.

**Figure 2:**
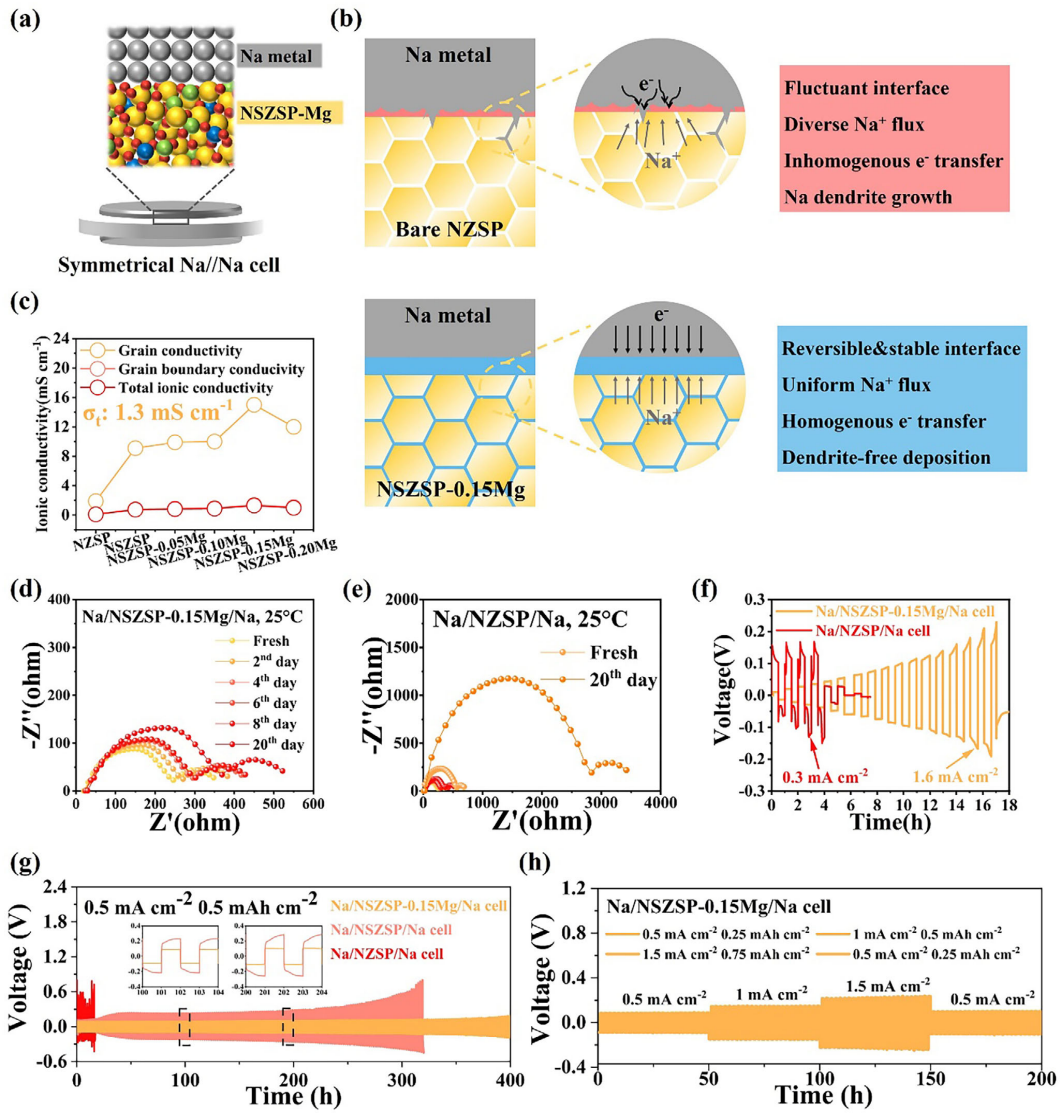
a) Schematic of the symmetrical Na//Na cell using NSZSP‐xMg as the solid‐state electrolyte. b) Schematic illustrations of the Na deposition behavior of the bare NZSP/Na interface and NSZSP‐0.15Mg/Na interface. c) Plot of ionic conductivities versus dopants. Time‐resolved Nyquist plots of (d) The Na//NZSP‐0.15Mg//Na cell and e) The Na//NZSP//Na cell measured at 25 °C. f) Critical current densities of NZSP and NSZSP‐0.15Mg. g) Galvanostatic cycling of Na//Na cell with NZSP, NSZSP, and NSZSP‐0.15Mg SSEs at a current density of 0.5 mA cm^−2^. h) Galvanostatic charge/discharge cycling profile of the Na//NZSP‐0.15Mg//Na cell under stepwise increased current densities.

To elucidate the enhancement in ionic conductivity of the NZSP SSEs resulting from the co‐doping strategy, the NZSP SSE pellets were gold‐sputtered on opposite faces and characterized using the Electrochemical Impedance Spectroscopy (EIS) method. The Nyquist plots of the Au//NZSP SSE//Au symmetric cells were collected by the EIS measurement at room temperature, and the results are shown in Figure S7a,b (Supporting Information). Basically, the Nyquist plots consist of a high‐frequency intercept on the x‐axis and one semicircle which are related with the bulk grain resistance (R_b_) and grain boundary resistance (R_gb_), respectively. An equivalent circuit is proposed in Figure S7c (Supporting Information) to determine the resistance values for each of the Au//NZSP SSE//Au symmetric cells. The bulk conductivity (σ_b_) and grain boundary conductivity (σ_gb_) can be calculated correspondingly, which together constitute the total ionic conductivity (σ_t_). The measured values have been included in Table S2 (Supporting Information). Specifically, the low σ_t_ of undoped NZSP SSE (0.1 mS cm^−1^) was heavily suppressed by a large total resistance (R_t_) (1125.8 Ω cm^−2^), which was caused by the voids and gaps observed from the SEM images. Impedance analysis reveals that the doping strategy of Sc^3+^ and Mg^2+^ effectively reduces both bulk grain and grain boundary resistance, thereby contributing to improved Na^+^ ion mobility, promoting correlated ion migration pathways, and significantly enhancing the ionic conductivity.^[^ ^21^ ^]^ Among the NZSP samples, NSZSP‐0.15Mg demonstrates the most favorable ionic conductivity with an R_t_ of only 89.5 Ω cm^−2^, achieving an optimal σ_t_ of 1.3 mS cm^−1^ at room temperature, in excess of one order of magnitude higher than that of undoped NZSP and around twice that of individual Sc^3+^‐doped NSZSP SSE, respectively. To investigate the migration barriers associated with ionic conduction at the interface between NZSP SSEs and Na metal, solid‐state Na//Na symmetric cells were assembled and evaluated with no external pressure, employing Na foils as electrodes and NZSP SSEs as the electrolyte (**Figure** **2**a). EIS measurement was conducted at room temperature to monitor the interfacial electrochemical behavior of symmetrical Na//Na cells, as reflected in Figure S8 (Supporting Information). Typically, the high‐frequency intercept on the x‐axis indicates the R_b_ of NZSP SSEs, the first arc corresponds to the R_gb_, and the low‐frequency arc represents the interface resistance (R_int_) at the Na/SSE interface. The resistances were extracted by fitting the impedance spectra using an equivalent circuit (Figure S7d, Supporting Information), and the specific values are listed in Table S3 (Supporting Information). A large R_t_ of 1710.5 Ω cm^−2^ was observed in the Na//NZSP//Na symmetrical cell, indicating the unsatisfied contact of Na metal with the undoped NZSP SSE. With an increasing amount of doped element, the R_t_ decreases correspondingly, while increasing again when the Mg^2+^ ion doping reaches 0.2, this trend is consistent with the previously obtained ionic conductivity results. The modified Na//NSZSP‐0.15Mg//Na symmetrical cell exhibits much better feasibility to ionic migration with an R_t_ of 348.1 Ω cm^−2^ at room temperature, which is around five times greater than that of the undoped one. A brief schematic illustration in Figure 2b shows the Na plating/stripping mechanism for the undoped NZSP SSE and optimal NSZSP‐0.15Mg SSE. For the bulk NZSP SSE, defects like voids and gaps induce the fluctuant Na/SSE interface, resulting in poor interfacial contact and inhomogeneous Na^+^ flux. The locally concentrated Na^+^ flux leads to a vertical nucleation tendency during continuous cycling, thereby causing the formation of Na dendrites. Additionally, the non‐uniform growth of Na dendrites leads to the release of localized stress and strain, causing the development of cracks on the SSE surface, which subsequently offers new channels for continued dendrite propagation.^[^ ^22^ ^]^ While for the optimal Sc^3+^/Mg^2+^ doped NZSP SSEs, a reversible and stable interface has been established owing to the novel co‐doping strategy, a uniform Na^+^ flux and homogenous e^−^ transfer can be effectively achieved owing to the enhanced ionic conductivity, which enables good interfacial contact and relieves the generated stress/strain. Therefore, the modified NSZSP‐Mg SSE with a dendrite‐free and stable interface will promote subsequent enhanced electrochemical performance. The σ_b_, σ_gb,_ and σ_t_ of all NZSP SSEs are shown in Figure 2c. Moreover, time‐resolved Nyquist plots of the symmetrical Na//Na cells are collected by EIS measurement at room temperature to monitor the interfacial electrochemical evolution. The specific values of resistances are shown in Table S4 (Supporting Information). As depicted in Figure 2d, the modified Na//NSZSP‐0.15Mg//Na symmetrical cell exhibits an adequate R_t_ of 367 Ω cm^−2^ at the fresh state and slightly increases to 551 Ω cm^−2^ after 20 days. Conversely, the bulk Na//NZSP//Na symmetrical cell shows an initial R_t_ of 689 Ω cm^−2^ and rapidly rises after 20 days to a value of 3444 Ω cm^−2^, indicating the interfacial degradation after storage (Figure 2e).

The critical current density (CCD) measurement was utilized through galvanostatic charge and discharge cycling at step‐wise increased currents to investigate how fast and safely the Na//Na symmetrical cells can run without forming dendrites or causing short‐circuit failure. As illustrated in Figure 2f, NSZSP‐0.15Mg achieves a notably high CCD of 1.6 mA cm^−2^, which is more than five times that of undoped bulk NZSP (0.3 mA cm^−2^). This significant enhancement reflects the improved tolerance of the Na//NSZSP‐0.15Mg//Na symmetrical cell to elevated current loads and the sufficient Na ion transfer kinetics at the interface. For comparison, CCD test results for other NZSP SSEs are presented in Figure S9 (Supporting Information). The respective CCD values for NSZSP, NSZSP‐0.05Mg, NSZSP‐0.10Mg, and NSZSP‐0.20Mg are 0.5, 0.6, 0.9, and 1.1 mA cm^−2^, demonstrating a consistent improvement upon Sc^3+^/Mg^2+^ doping. Galvanostatic discharge/charge cycling of the Na//Na symmetrical cells was conducted to elucidate the interfacial stability during the repeated Na plating/stripping cycles. As depicted in Figure 2g, the Na//NSZSP‐0.15Mg//Na symmetrical cell delivers a superior cycling performance for over 400 h at 0.5 mA cm^−2^, indicating the effectiveness of the co‐doping strategy on suppressing the interfacial resistance. While for the single Sc^3+^‐doped NSZSP SSE, the symmetrical cell undergoes fluctuating and irregular voltage curves, a polarization voltage occurs after cycling for 300 h. The undoped NZSP SSE, on the other hand, shows a sharp polarization and a quick short circuit within the cycling time for only 20 h, revealing poor Na metal plating/stripping cycling stability. Figure S10 (Supporting Information) shows the Na metal plating/stripping cycling profiles of the Na//Na symmetrical cells using NSZSP‐0.05Mg, NSZSP‐0.10Mg, and NSZSP‐0.20Mg SSEs, respectively. Additionally, a rate cycling of the Na//NSZSP‐0.15Mg//Na symmetrical cell under stepwise increased current densities of 0.5, 1, 1.5 mA cm^−2^ was performed, which maintains a stable Na metal plating/stripping for over 300 h (Figure 2h).

To gain deeper insight into the ion transport mechanism of the Sc^3+^/Mg^2+^ doped NZSP SSEs with enhanced ionic conductivities, X‐ray absorption spectroscopy (XAS) measurements at the P, Si, Zr, Sc, Mg, and O K‐edges were employed to probe the local chemical environments and electronic states of the elements within the conduction band of the NSZSP‐xMg SSEs. The K‐edge X‐ray absorption near edge structure (XANES) refers to the region of the absorption spectrum within 30–50 eV near the absorption edge energy, electronic transitions originating from the 1s core level of the absorbing atom to unoccupied states of appropriate symmetry, primarily involving p‐orbital character, in accordance with dipole selection rules.^[^ ^23^ ^]^ Consequently, the energy position of the absorption edge (edge jump threshold) and the intensity of the associated peak (commonly referred to as the white line) are highly sensitive to the local chemical environment of the absorbing species. These spectral features are typically interpreted through comparison with reference standards of known structure and composition. Specifically, the normalized P K‐edge XANES spectra of NSZSP‐xMg SSEs with varying Mg^2+^ doping levels are presented in Figure S11a (Supporting Information). All samples exhibit a pronounced white line feature centered at ≈2153.2 eV, corresponding to the excitation of P 1s electrons into unoccupied states formed by the overlap of P sp^3^ hybrid orbitals and O 2p orbitals, consistent with the tetrahedral symmetry of the PO_4_3^−^ tetrahedra.^[^ ^24^ ^]^ The consistent shape and position of the white line and post‐edge feature across all samples suggest that Sc^3+^/Mg^2+^ co‐doping does not disrupt the local PO_4_3^−^ geometry. A broader peak observed near 2169 eV is attributed to multiple scattering events involving first‐shell oxygen atoms in the PO_4_^3−^ units. The similarity in spectral shape and edge position across all samples indicates that Mg doping does not significantly alter the local coordination environment of P, suggesting that the phosphate substructure remains structurally robust. As shown in the magnified view (Figure S11b, Supporting Information), minor variations in the white line intensity and slight energy shifts, particularly in NSZSP‐0.15Mg and NSZSP‐0.20Mg samples, suggest subtle changes in the NASICON structure and local bonding environment around P atoms. These spectral differences may be associated with enhanced ionic transport properties resulting from Mg‐induced modification of the surrounding lattice. For Si K‐edge (Figure S11c, Supporting Information), the high degree of overlap in both spectral shape and energy position across the different doping concentrations indicates that Mg incorporation does not significantly perturb the local coordination environment of Si atoms. Only minor variations in white line intensity are observed in Figure S11d (Supporting Information), attributing to subtle changes in the electronic structure or local lattice distortions induced by aliovalent doping. Importantly, the absence of new absorption features confirms that the SiO_4_ tetrahedral framework remains chemically and structurally intact upon Sc^3+^/Mg^2+^ doping.

**Figure 3:**
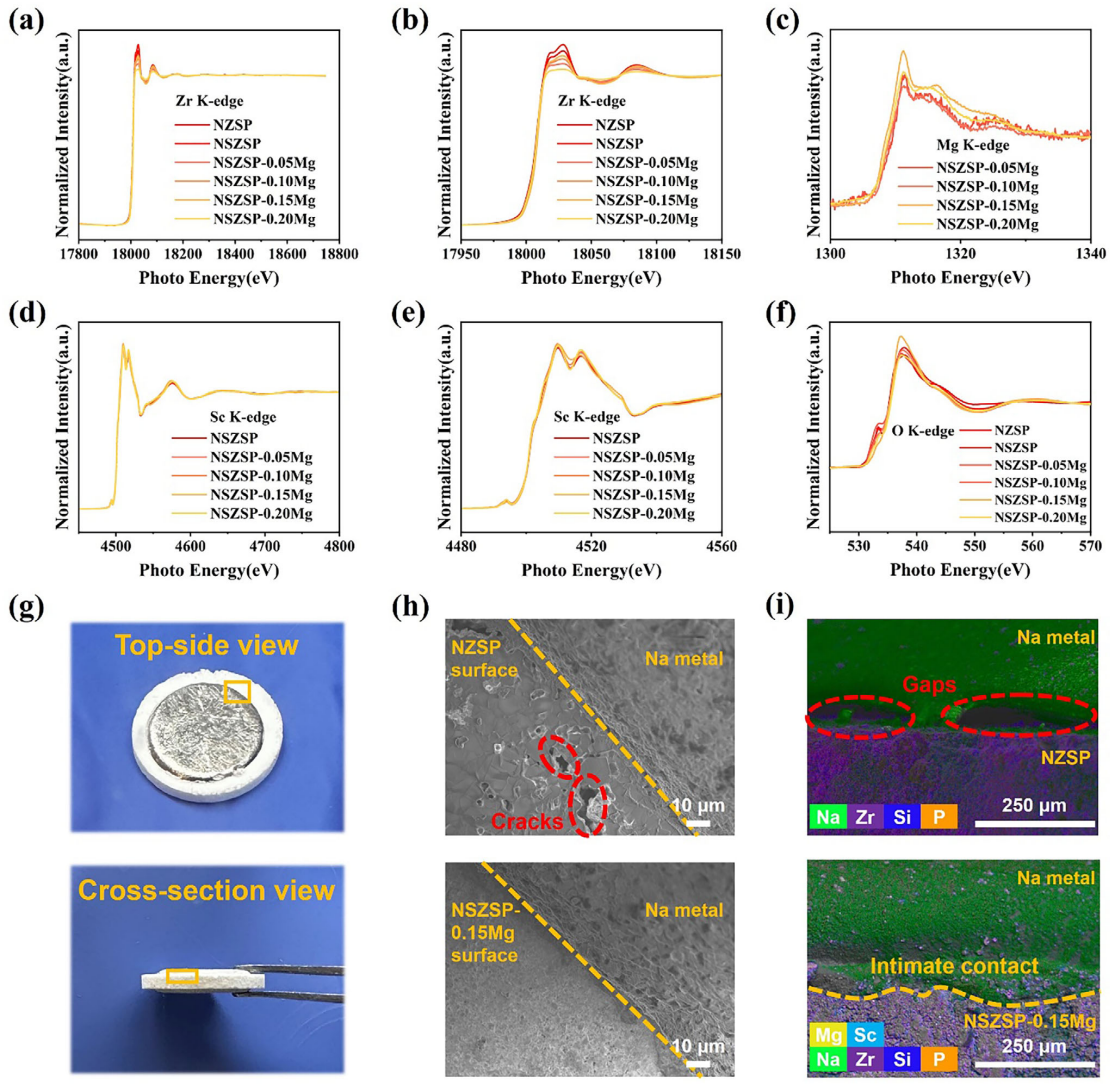
a) Zr K‐edge XANES spectra for NSZSP‐xMg and b) a magnified view of the selected energy region in Zr K‐edge spectra. c) Mg K‐edge XANES spectra for NSZSP‐xMg. d) Sc K‐edge XANES spectra for NSZSP‐xMg and e) a magnified view of the selected energy region in Sc K‐edge spectra. f) O K‐edge XANES spectra for NSZSP‐xMg. g) Schematics of top‐side and cross‐section view of the after‐cycled Na‐capped NSZSP‐0.15Mg SSE pellet. h) Top‐side view SEM images and i) cross‐section view SEM‐EDS mapping images of the NZSP and NSZSP‐0.15Mg against Na metal after cycling.

Zr K‐edge XANES spectra of the NSZSP‐xMg SSEs (**Figure** **3**a,b) offer critical insight into the local electronic and structural modifications induced by partial substitution of Zr^4+^ with Sc^3+^/Mg^2+^ ions. Across all samples, the primary absorption edge appears consistently ≈18 020 eV, indicative of Zr^4+^ oxidation state and suggesting a slight reduction in the effective oxidation state of Zr upon Sc^3+^/Mg^2+^ co‐doping, which could lower the coulombic repulsion between the doped ions and Na^+^ ions, and requires less energy to navigate through the bottlenecks around the Zr^4+^ ions.^[^ ^21^, ^25^ ^]^ Subsequently, with increasing Mg doping concentration, the white line intensity becomes progressively diminished and slightly broadened, which reflects a reduction in the density of unoccupied Zr 5d states. This effect can be attributed to substitutional doping at the Zr site, where the incorporation of Sc^3+^/Mg^2+^ co‐doping induces local lattice distortions and modifies the Zr─O coordination environment, leading to subtle variations in electronic structure. Furthermore, the observed changes in post‐edge oscillations imply minor rearrangements in the local bonding geometry surrounding the residual Zr atoms. Collectively, these spectral features confirm that while the NASICON framework remains structurally stable, the local electronic environment around Zr is perturbed by Sc^3+^/Mg^2+^ co‐doping, consistent with aliovalent substitution and the accompanying charge compensation mechanisms.^[^ ^26^ ^]^ The Sc K‐edge XANES spectra of the NSZSP‐xMg SSEs reveal systematic changes in the local electronic and structural environment around Sc. As shown in Figure 3d,e, the gradual shift in the main absorption edge and subtle variations in the pre‐edge features indicate modifications to the electronic structure and coordination geometry of Sc^3+^ upon Mg^2+^ doping, which optimize the coordination environment of Sc by making the Sc─O octahedra more regular or uniform. When the Mg^2+^ doping content reaches 0.15, the local structure appears to be most ideal, and the cooperation effect induced by doping is most obvious, resulting in an increased absorption cross‐section and the highest white line intensity for Sc. However, excessive Mg^2+^ doping may destroy this local order, potentially introducing new structural defects or coordination mismatches, thereby reducing the local symmetry or affecting the electronic state of Sc, which leads to a decrease in absorption intensity. The Mg K‐edge results in Figure 3c are consistent with the previous observations for Sc K‐edge, indicating that NSZSP‐0.15Mg sample represents the optimal structure. As the doping amount increases, Mg^2+^ ions become more uniformly distributed within the lattice, and their coordination environment tends to be stable. This structural refinement enhances the X‐ray absorption response of Mg, leading to an increase in normalized intensity. At this co‐doping ratio, Na^+^ ions can navigate more effectively through the bottlenecks, thereby improving Na transport and ultimately enhancing the ionic conductivity. The O K‐edge XAS spectra are illustrated in Figure 3f. The pre‐edge region corresponds to transitions from O 1s to O 2p orbitals hybridized with neighboring metal orbitals. NSZSP‐0.15Mg exhibits an optimal balance in pre‐edge intensity, indicating favorable hybridization between O 2p states and the orbitals of Sc, Mg, Zr, and Si. The results of the main absorption peak ≈535–545 eV are complemented, where NSZSP‐0.15Mg shows enhanced intensity and minimal broadening, suggesting an electronic structure that promotes stability and efficient ionic conduction. The post‐edge region further supports this observation, with NSZSP‐0.15Mg displaying well‐defined oscillatory features, indicative of reduced lattice distortions and an improved medium‐range structural order. The superior structural and electronic features of NSZSP‐0.15Mg are expected to enhance Na‐ion diffusion pathways, resulting in improved ionic conductivity and overall performance. The XANES analysis, in agreement with XRD, SEM, and EDS mapping results, confirms the successful incorporation of partial substitution of Zr^4+^ with Sc^3+^/Mg^2+^ ions in NSZP structure.

To clarify the interface interaction and interfacial chemistry between NSZSP‐xMg and Na metal, cross‐sectional SEM images along with corresponding EDS images of the Na//Na symmetrical cells post cycling are investigated. Figure 3g illustrates the top‐side and cross‐section view of the disassembled Na‐capped NSZSP‐0.15Mg SSE pellet after cycling, showing that the Na metal is in intimate contact with the SSE pellet after cycling with no obvious short circuit breakdown or Na metal dendrites. The corresponding top‐side view SEM images and cross‐section view SEM‐EDS mapping images of the NZSP and NSZSP‐0.15Mg against Na metal plating/stripping are revealed in Figure 3h,i, where critical defects like cracks and gaps can be detected on the loose NZSP SSE surface and between the fluctuating SSE/Na interface, respectively. These shortcomings ultimately lead to uneven Na metal plating and promote dendritic growth. Impressively, Na metal remains well‐adhered to the denser surface of NSZSP‐0.15Mg SSE, and no evidence of Na dendrite formation is observed across the NSZSP‐0.15Mg, which continues to maintain intimate interfacial contact with the Na metal even after prolonged charge/discharge cycling. Figure S12 (Supporting Information) provides the details of each element of the SEM‐EDS mapping images. These findings indicate that the incorporation of Sc/Mg species into NZSP significantly enhances a beneficial interphase layer, effectively facilitating homogeneous Na metal plating/stripping at the contact interface and improving the chemical compatibility of the SSE with Na metal.

**Figure 4:**
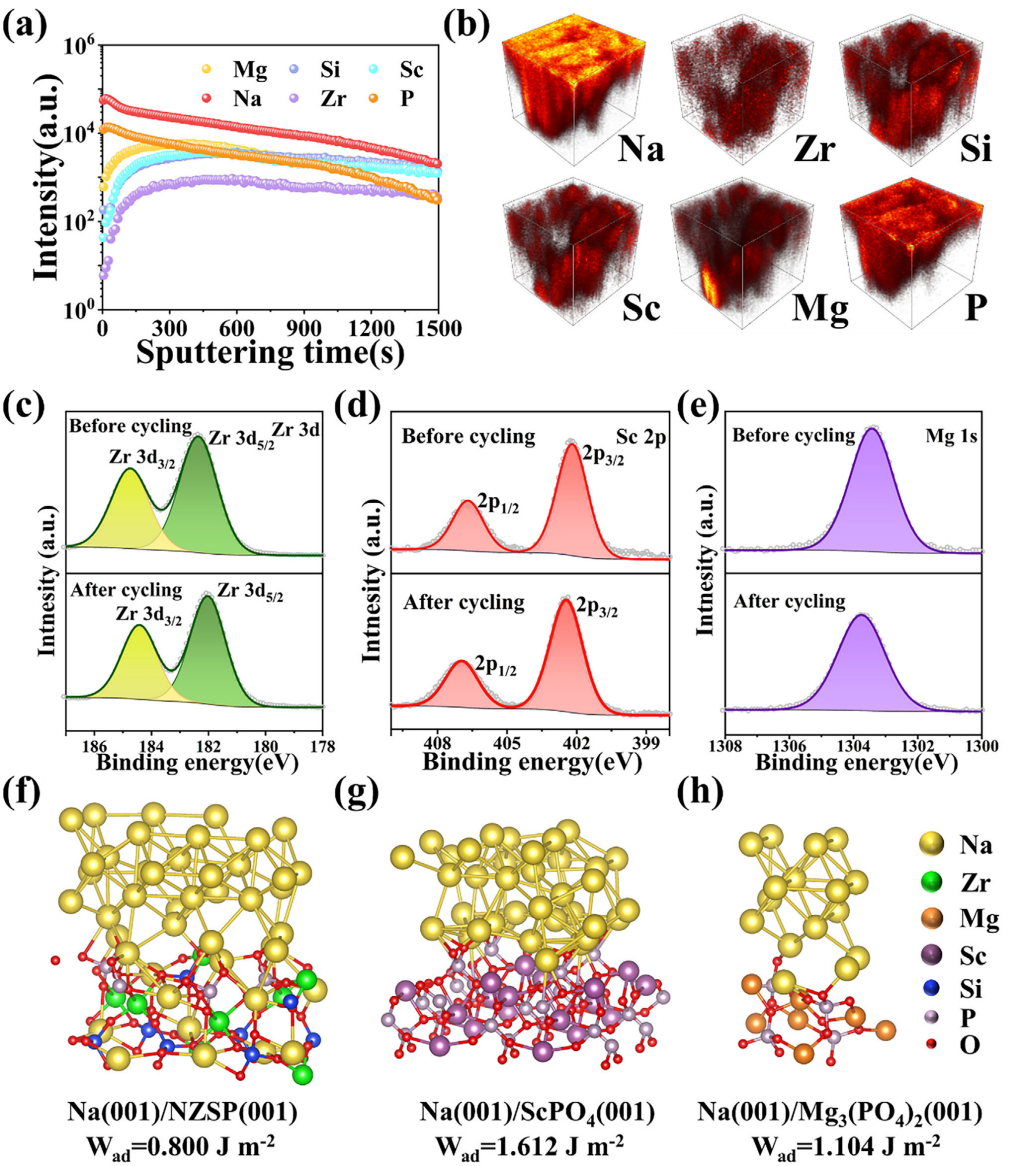
a) In‐depth ToF‐SIMS profile of NSZSP‐0.15Mg surface after Na plating/stripping cycles, and b) the corresponding 3D model images of Na, Zr, Si, Sc, Mg, and P species. XPS spectra of NSZSP‐0.15Mg before and after Na stripping/plating cycling: c) Zr 1s; d) Sc 2p; e) Mg 1s. Simulated work of adhesion (W_ad_) at the interface of f) Na/Na_3_Zr_2_Si_2_PO_12_, g) Na/ScPO_4,_ and h) Na/Mg_3_(PO_4_)_2_.

Time‐of‐flight secondary ion mass spectrometer (ToF‐SIMS) was performed using an Ar^+^ beam (1 kV, 300 nA) on a sputtering area of 300 µm × 300 µm to analyze the interfacial condition following Na metal plating. As shown in **Figure** **4**a, considering the nature of the ToF‐SIMS detection process and sample morphology changes during sputtering, the depth profiles reveal stable counting rates for Zr and Si throughout the sputtering process, suggesting that their concentration remains relatively constant, supporting the interpretation of compositional stability at deeper regions of NZSP SSEs. This finding is consistent with XAS results. In contrast, signals for Na, Sc, Mg, and P decrease with increasing sputtering time and depth, indicating surface enrichment of these elements in the NSZSP‐0.15Mg sample. The corresponding 3D in‐depth model images in Figure 4b further illustrate the formation of an interphase layer dominated by Na‐Sc‐Mg‐P‐O species between Na metal and SSE. This layer is chemically stable against highly reactive Na, thereby preventing further interfacial degradation or undesirable side reactions, which facilitates improved interfacial performance by providing a fast ion transport channel that supports smooth Na metal plating/stripping. Moreover, this interphase acts as a chemically compatible and mechanically resilient buffer layer, mitigating stress accumulation and suppressing interfacial fracture or delamination during cycling.^[^ ^27^ ^]^ Additional ToF‐SIMS analysis was collected on the Na//NSZSP‐0.15Mg//Na symmetrical cell before cycling. As shown in Figure S13 (Supporting Information), all elements are steady with the increasing sputtering times, indicating the NSZSP‐0.15Mg sample is well synthesized with a uniform distribution of elements. Figure S14 (Supporting Information) illustrates the integrated images of the NSZSP‐0.15Mg surface before and after Na metal plating/stripping cycles, which agree with the ToF‐SIMS observation.

X‐ray photoelectron spectroscopy (XPS) was employed to analyze the changes in chemical states and to perform spontaneous characterization of NSZSP‐0.15Mg before and after cycling. Typical Mg 1s, Na 1s, O 1s, Sc 2p, Zr 3d, P 2p, Si 2p species with the C 1s calibration are observed in the survey XPS spectra (Figure S15, Supporting Information). Specifically, Figure 4c shows the high‐resolution XPS spectrum of Zr 3d, where a characteristic Zr^4+^ state corresponding to Zr^4+^(PO_4_)^3−^ is observed before cycling. The binding energy of Zr 3d gets reduced after cycling, verifying the formation of a low‐valence Zr^(4−x)+^ state. Both the slight shifting of Sc 2p and Mg 1s species to high binding energy after cycling are demonstrated in Figure 4d,e, implying the formation of interfacial reaction products including Na‐Sc/Mg‐O and Na‐Sc/Mg‐P‐O species, indicating a chemically altered interphase layer at the interface between Na metal and SSE. Simultaneously, decreased Na KLL peak and Si‐O peak with increased Mg‐O peak in the O 1s spectrum (Figure S16a, Supporting Information) further confirms the aggregation of Na species on the surface after cycling. Na 1s, Si 2p, P 2p peaks are detected both before and after cycling (Figure S16b–d, Supporting Information). These findings align with the ToF‐SIMS analysis that a dynamically stable interphase layer induced by the ScPO_4_/Mg_3_(PO_4_)_2_‐dominant species were formed between Na metal and NSZSP‐0.15Mg SSE. To unravel the microscopic mechanisms by which such an interphase improves interfacial contact, we conducted density functional theory (DFT) computations to compare the work of adhesion (W_ad_) between Na_3_Zr_2_Si_2_PO_12_, ScPO_4_, Mg_3_(PO_4_)_2,_ and Na metal.^[^ ^12^, ^14^, ^28^ ^]^ As demonstrated in Figure 4f–h, the calculated W_ad_ values for the three interface models are 0.800, 1.612, and 1.104 J·m^−2^, respectively. The significantly higher adhesion energies associated with the ScPO_4_ and Mg_3_(PO_4_)_2_ surfaces indicate a substantial enhancement in interfacial wettability and bonding with Na metal, facilitated by the formation of an interphase layer. The exceptional interfacial behavior of the NSZSP‐0.15Mg composition can be largely attributed to its dense microstructural features and favorable interfacial reaction characteristics with Na metal. These findings are consistent with ToF‐SIMS analysis.

**Figure 5:**
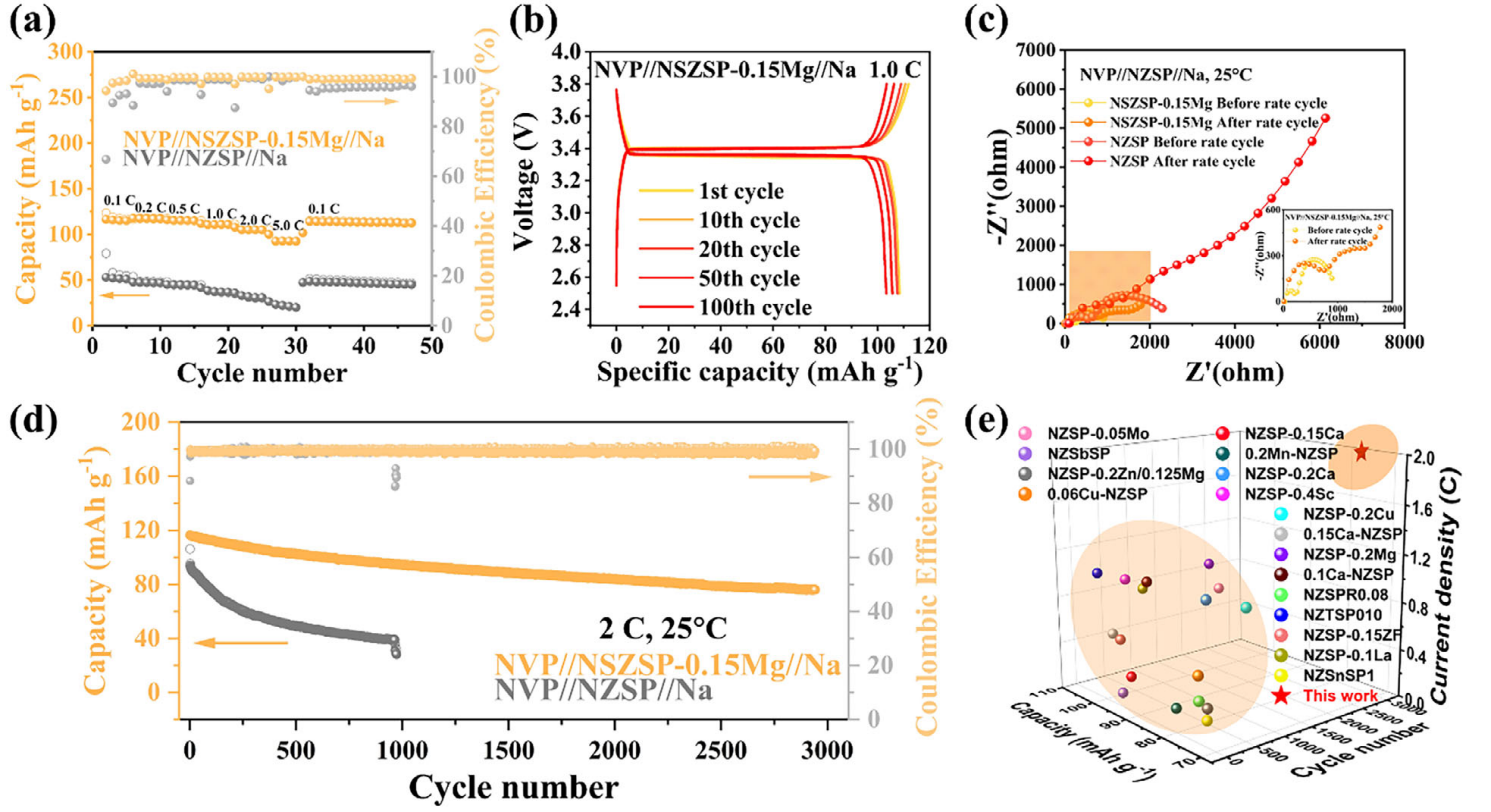
a) Rate performance in the range of 0.1‐5 C measured at room temperature. b) Galvanostatic charge/discharge cycling profile for the selected cycles at the rate of 0.1 C. c) Nyquist plots before and after the rate cycling. d) Long‐cycling performance at 2 C for 3000 cycles at room temperature. e) Cycling performance comparison between NSZSP‐0.15Mg and other NZSP‐based solid electrolytes.

To assess the practical applicability of NSZSP‐0.15Mg as a high‐performance and flexible SSE, CR2032‐type solid‐state full cells were assembled using a typical NASICON‐type cathode material of Na_3_V_2_(PO_4_)_3_ (NVP), NSZSP‐xMg SSEs, and Na‐metal foil anode. To improve interfacial contact between NVP and SSE, 10 µL of the liquid electrolyte (1.0 M NaClO_4_ in EC/DEC (1:1) and 5% FEC) was added to the cathodic side as a wetting agent.^[^ ^29^ ^]^ As depicted in **Figure** **5**a, the optimized NVP//NSZSP‐0.15Mg//Na cell exhibits excellent rate performance across stepwise increased current densities from 0.1 to 5 C (1 C ≈ 117.6 mA g^−1^) every 5 cycles. An impressive charge/discharge capacity of 123.4/116.4 mAh g^−1^ at the first cycle with a Coulombic efficiency of 94.3% at 0.1 C can be achieved. Even at the high rate of 5 C, a reversible capacity of 92.4 mAh g^−1^ is retained. Notably, when the current density is reset to 0.1 C after cycling at 5 C for 5 cycles, the charge/discharge profiles closely match the earlier cycles at 0.1 C, delivering a stable reversible capacity of 115 mAh g^−1^.

Figure S17a (Supporting Information) compares the rate performance of the NVP//Na cells using the NSZSP‐0.05Mg, NSZSP‐0.10Mg, and NSZSP‐0.20Mg SSEs. In contrast, the unmodified NVP//NZSP//Na cell exhibits poor rate performance, achieving only 52 mAh g^−1^ at 0.1 C, highlighting the interfacial advantages of the doped NSZSP‐0.15Mg SSE. Galvanostatic charge/discharge cycling profile of the NVP//NSZSP‐0.15Mg//Na cell at 1.0 C is illustrated in Figure 5b with the selected 1st, 10th, 20th, 50th, and 100th cycles in the voltage window of 2.5–3.8 V versus Na^+^/Na, where a good overlap of the typical charge/discharge curves and an initial discharge capacity of 111.9 mAh g^−1^ are delivered. Galvanostatic charge/discharge cycling profiles of other SSEs are listed in Figure S18 (Supporting Information). Simultaneously, EIS measurements before and after rate cycling are performed at room temperature to investigate the electrochemical processes in the NVP//Na cells. These semicircles in the Nyquist plots are associated with the solid electrolyte (R_SE_), the anode interfacial charge transfer (R_ct_), and the cathode interfacial charge transfer (R_cathode_). As shown in Figure 5c and Table S5 (Supporting Information), for the NVP//NZSP//Na cell, which shows a large R_ct_ of 533 Ω cm^−2^ and increases to 1052 Ω cm^−2^ after rate cycling. While for the optimal NVP//NSZSP‐0.15Mg//Na cell, an adequate R_ct_ of 209 Ω cm^−2^ can be delivered, indicating a significantly enhanced interfacial charge transfer and strong compatibility between the SSEs and the NVP cathode. The long‐cycling performance of the solid‐state NVP//Na cells at a constant rate of 2.0 C is exhibited in Figure 5d, where a discharge capacity of 75 mAh g^−1^ of the optimal NVP//NSZSP‐0.15Mg//Na cell is achieved with a capacity retention of 64% over 3000 cycles at room temperature. The formation of the ScPO_4_/Mg_3_(PO_4_)_2_‐rich interphase layer stabilizes the interface by creating an intimate and low‐impedance interfacial contact between Na and SSEs, facilitating fast interfacial charge‐transfer kinetics and thereby suppressing pore or void formation to inhibit dendrite growth. Without any doping optimization, the undoped NVP//NZSP//Na cell exhibits capacity fading and unsatisfactory cycling stability, which only delivers a 40 mAh g^−1^ during the cycling process. The poor electrochemical performance could be attributed to the gradual deterioration at the NZSP SSE/electrodes interface and the buildup of internal polarization. Figure S17b (Supporting Information) shows the cycle performance of the NVP//Na cells using the NSZSP‐0.05Mg, NSZSP‐0.10Mg, and NSZSP‐0.20Mg SSEs. The long‐cycling results closely align with those observed in symmetrical Na//Na cells, further confirming that the stable cycling performance stems from the highly compatible and conductive interface at SSE/electrodes interface, thereby maintaining long‐term chemical stability and mechanical robustness. Figure 5e presents a comparison between the NSZSP‐0.15Mg SSE developed in this study and previously reported NZSP‐based SSEs, with detailed information on cycle number, current density, and capacity in full cells summarized in Table S6 (Supporting Information). Clearly, the optimized NSZSP‐0.15Mg SSE is superior among the NZSP‐based SEs, highlighting the effectiveness of the Sc^3+^/Mg^2+^ co‐doping strategy on enhancing the ionic conductivity and promoting the interfacial performance.

Taking all these outstanding electrochemical performances into account, the remarkable rate performance and long‐cycling stability under high current density are expected to enable rapid energy conversion and efficient storage, making NSZSP‐0.15Mg SSE a promising candidate in energy storage systems. Thus, the application of NZSP‐based SSEs with novel co‐doping strategy holds great potential to fulfill the demands of solid‐state sodium metal batteries for large‐scale energy storage applications in future commercial markets.

## 3 Conclusion

In summary, we demonstrate a novel Sc^3+^/Mg^2+^ co‐doping strategy to significantly enhance ionic conductivity in SSEs by directly synthesizing Na_3.7_Zr_1.45_Sc_0.4_Mg_0.15_Si_2_PO_12_ (NSZSP‐0.15Mg) via a facile solid‐state reaction method. The Mg content is rationally controlled to regulate the interfacial chemistry. Specifically, the incorporation of flexible Sc^3+^ and Mg^2+^ ions facilitates Na^+^ migration, resulting in an enhanced ionic conductivity of 1.3 mS cm^−1^ at room temperature. A galvanostatic Na//Na symmetrical cell employing the optimal NSZSP‐0.15Mg exhibits ultra‐stable Na plating/stripping over 400 h at 0.5 mA cm^−2^, highlighting improved electrochemical kinetics. XAS measurements probed the local and electronic structures of the constituent elements, while ToF‐SIMS analysis and first‐principles calculations confirm that performance improvements stem from a favorable interphase layer dominated by ScPO_4_ and Mg_3_(PO_4_)_2_, induced by the Sc^3+^/Mg^2+^ co‐doping strategy. The high performance is further validated in a Na//Na_3_V_2_(PO_4_)_3_ full cell, which delivers a capacity of 75 mAh g^−1^ at 2.0 C over 3000 cycles, with excellent rate capability at room temperature. This work provides significant insights into the development of solid‐state Na‐batteries with high energy density, safety, and long‐term stability, establishing a promising foundation for their practical application in the growing renewable energy market.

## 4 Experimental Section

### Materials

The raw materials sodium carbonate (Na_2_CO_3_), zirconium (IV) oxide (ZrO_2_), silicon dioxide (SiO_2_), scandium (III) oxide (Sc_2_O_3_), magnesium oxide (MgO), and ammonium phosphate monobasic (NH_4_H_2_PO_4_) were purchased from Sigma–Aldrich and used as received without further purification. Na_3_V_2_(PO_4_)_3_ (NVP), sodium perchlorate (NaClO_4_), ethylene carbonate (EC), diethyl carbonate (DEC), and fluoroethylene carbonate (FEC) were purchased from Sigma–Aldrich and used as received without further purification. Carbon black, polyvinylidene fluoride (PVDF), and N‐methyl‐2‐pyrrolidone (NMP) were purchased commercially. The batteries (CR‐2032 type) were purchased from Saibo Electrochemical Materials (Taobao, China).

### Preparation of the Solid‐State Electrolytes

NASICON solid‐state electrolytes with nominal compositions of Na_3_Zr_2_Si_2_PO_12_, Na_3.4_Zr_1.6_Sc_0.4_Si_2_PO_12_, Na_3.5_Zr_1.55_Sc_0.4_Mg_0.05_Si_2_PO_12_, Na_3.6_Zr_1.5_Sc_0.4_Mg_0.1_Si_2_PO_12_, Na_3.7_Zr_1.45_Sc_0.4_Mg_0.15_Si_2_PO_12_, Na_3.8_Zr_1.4_Sc_0.4_Mg_0.20_Si_2_PO_12_ were synthesized by a standard solid‐state synthesis route.^[^ ^30^ ^]^ High‐purity raw materials Na_2_CO_3_, ZrO_2_, SiO_2_, Sc_2_O_3_, MgO, and NH_4_H_2_PO_4_ were mixed in their respective stoichiometric ratios. Na_2_CO_3_ and NH_4_H_2_PO_4_ were used with a 10 at% excess to compensate for their volatility at high temperatures. The raw materials, dispersed in 20 mL of isopropanol, were mixed using a planetary ball mill at 300 rpm overnight to form the precursor. The resulting mixture was then dried at 80 °C. Subsequently, the dried precursor was pre‐sintered in air at 1100 °C for 8 h with a ramp rate of 2 °C/min. The calcined powder was milled at 300 rpm for 12 h to achieve a homogeneous state, and then pressed into pellets using a 14 mm steel die. Finally, the pellets were sintered in an alumina boat at 1100 °C for 8 h with a sufficient layer of the original calcined powder as a bed to suppress sodium loss. After sintering, the pellets were polished with silicon carbide abrasive papers of varying grit sizes (#1000, #2000, #3000, #5000) and then ultrasonicated for 5 min in ethanol. The final pellets had a thickness of ≈0.9 mm and a diameter of ≈13 mm.

### Characterization

The crystal phase of the synthesized solid‐state electrolyte samples was analyzed using powder X‐ray diffraction (XRD) on a PANalytical Aeris X‐ray diffractometer with Cu Kα radiation (λ = 1.5406 Å) at a scanning rate of 2°/min. Rietveld refinement was performed using GSAS software on XRD patterns recorded over a 2θ range from 10 to 70°, with a count time of 2 s per step and a step size of 0.02°. Morphology, structural features, and atomic‐scale images were characterized by scanning electron microscopy (SEM, JEOL 7500) and transmission electron microscopy (TEM, JEOL JEM‐2011 Limited Corporation, Japan), respectively. X‐ray photoelectron spectroscopy (XPS) was performed on a Thermo Scientific Nexsa spectrometer to study surface chemical states. Time‐of‐Flight Secondary Ion Mass Spectrometry (ToF‐SIMS) was used for depth profiling using an Ar^+^ beam (1 kV, 300 nA) for sputter etching. The analysis area was 100 µm × 100 µm within the sputtering area of 300 µm × 300 µm. For XPS and ToF‐SIMS, samples were transferred via a transition chamber connected to an Ar‐filled glove box to avoid ambient air pressure. Synchrotron X‐ray absorption spectroscopy (XAS) measurements were conducted at the XAS, MEX‐1, MEX‐2, and SXR beamlines at the Australian Synchrotron (ANSTO) in Melbourne.

### Electrochemical Measurements

The solid‐state electrolyte pellets were coated with gold via ion beam sputtering to create blocking electrode contacts for ionic conductivity measurements. The ionic conductivity (σ) was calculated using σ = L/RS, where L is the thickness of the separator, R is the electronic resistance of the electrode, and S is the electrode area. Electrochemical impedance spectroscopy (EIS) measurements of the solid‐state electrolytes and all cells were performed using a Biologic VMP3 electrochemical workstation. Symmetric Na//NASICON//Na cells were assembled in an Ar‐filled glovebox (O_2_ < 0.1 ppm; H_2_O < 0.1 ppm) by directly placing Na metal foils on both sides of the solid‐state electrolyte pellets. The critical current density (CCD) of the symmetric cells was tested by galvanostatic charge and discharge, starting from 0.1 mA cm^−2^ with an increasing speed of 0.1 mA cm^−2^. Galvanostatic cycling of the symmetrical cells was conducted on a multi‐channel battery test system at 25 °C using a temperature‐controlled incubator. Solid‐state sodium metal batteries were assembled using Na_3_V_2_(PO_4_)_3_ (NVP) as the cathode, sodium metal as the anode, and NASICON samples as the solid‐state electrolyte. NVP powder, carbon black, and polyvinylidene fluoride (PVDF) in a weight ratio of 7:2:1 were mixed with N‐methyl‐2‐pyrrolidone (NMP) to obtain a uniform slurry. The resulting slurry was then cast onto an aluminum foil current collector and dried for 12 h under vacuum at 90 °C. The coated aluminum foil was punched into discs with a diameter of 6 mm and an active mass loading of 1.5 mg cm^−2^. 10 µL of liquid electrolyte (1.0 Mm NaClO_4_ in EC/DEC (1:1) with 5% FEC) was added to boost the Na^+^ transportation between the cathode and the solid electrolyte. The galvanostatic cycling of the sodium full cells was characterized in the voltage range of 2.5‐3.8 V versus Na/Na^+^ at 25 °C using a Neware battery test system.

### Density Functional Theory (DFT)

All the DFT calculations were performed using OpenMX package with Perdew‐Burke‐Ernzerhof (PBE) exchange‐correlation functional.^[^ ^31^ ^]^ Standard‐level basis sets were used for the expansion of the Kohn‐Sham orbitals.^[^ ^32^ ^]^ To describe the core electrons of atoms, MBK norm‐conserving pseudopotentials were employed.^[^ ^33^ ^]^ A Monkhorst‐Pack k‐point mesh density of 0.04 Å^−1^ was used, with Fermi smearing of 300 K.^[^ ^34^ ^]^ The (001) surfaces of Na_3_Zr_2_Si_2_PO_12_, ScPO_4_, and Mg_3_(PO_4_)_2_, were coupled with Na (001) to construct interface models; the slab thickness was set to ≈ 12 Å. Wavefunctions were relaxed until the electronic energy change was below 10^−6^ Hartree, and structures were relaxed until the maximum force on each atom was below 0.02 eV/Å. The work of adhesion (W_ad_) was defined as follows: W_ad_ = (E_Na_+E_substrate_−E_Na/substrate_)/A, where E_Na_, E_substrate_, and E_Na/substrate_ represent the energies of the Na (001) surface, the substrate, and the Na/substrate interface, respectively, and A is the interfacial area. The ASE and VESTA packages were used to set up and analyze the calculations.^[^ ^35^ ^]^

## Conflict of Interest

The authors declare no conflict of interest.

## Supporting information



Supporting Information

## Data Availability

The data that support the findings of this study are available from the corresponding author upon reasonable request.
