# Supplementary material for: Sc/Mg Co‐Doping in Na3Zr2Si2PO12 Solid‐State Electrolytes Enables Outstanding Performance of Sodium Metal Batteries
Source: Adv Sci (Weinh). 2025 Sep 24;12(46):e15463. doi: 10.1002/advs.202515463 (PMC12697781; doi:10.1002/advs.202515463)
Supplement: Supplementary file 1 — Supporting Information [file ADVS-12-e15463-s001.docx]

Supporting Information

Sc/Mg co-doping in Na_3_Zr_2_Si_2_PO_12_ Solid-state Electrolytes Enable Outstanding Performance of Sodium Metal Batteries

Xin Wang, Jiayang Li, Zewei Hu, Xinghan Li, Liyang Liu, Jiazhao Wang, Jung Ho Kim, Weijie Li*, Wei Kong Pang*, and Bernt Johannessen*

X. Wang, J. Li, X. Li, J. H. Kim, W. K. Pang, B. Johannessen

Faculty of Engineering and Information Sciences, University of Wollongong

NSW 2500, Australia
E-mail: wkpang@uow.edu.au

B. Johannessen

Australian Synchrotron, ANSTO, Clayton

VIC 3168, Australia
E-mail: berntj@ansto.gov.au

Z. Hu, L. Liu, W. Li

State Key Laboratory for Powder Metallurgy, Central South University

Changsha 410083, China
E-mail: li-306@csu.edu.cn

J. Wang

Wenzhou University Technology Innovation Institute for Carbon Neutralization

Wenzhou 325035, China


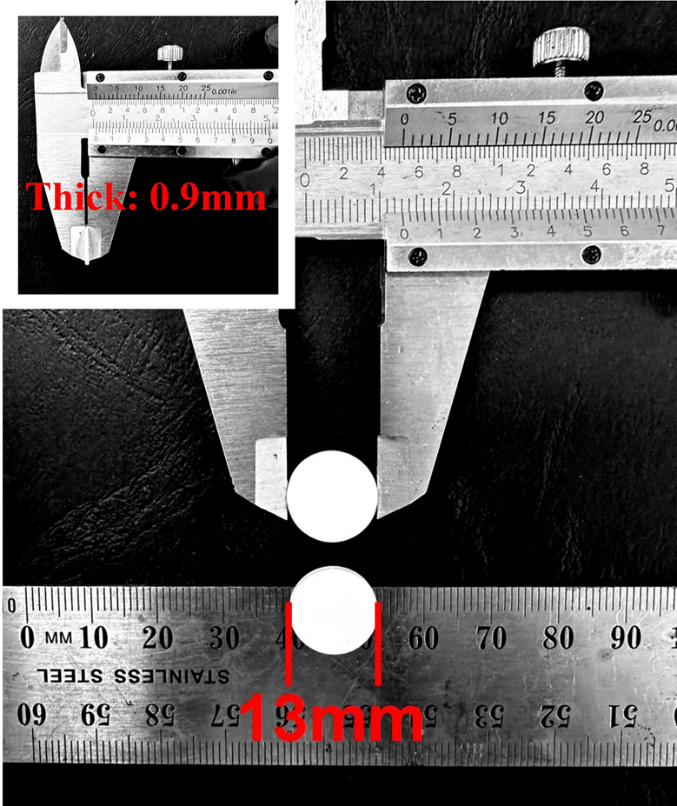


**Figure S1.** Diameter and thickness of obtained NZSP SSEs pellets


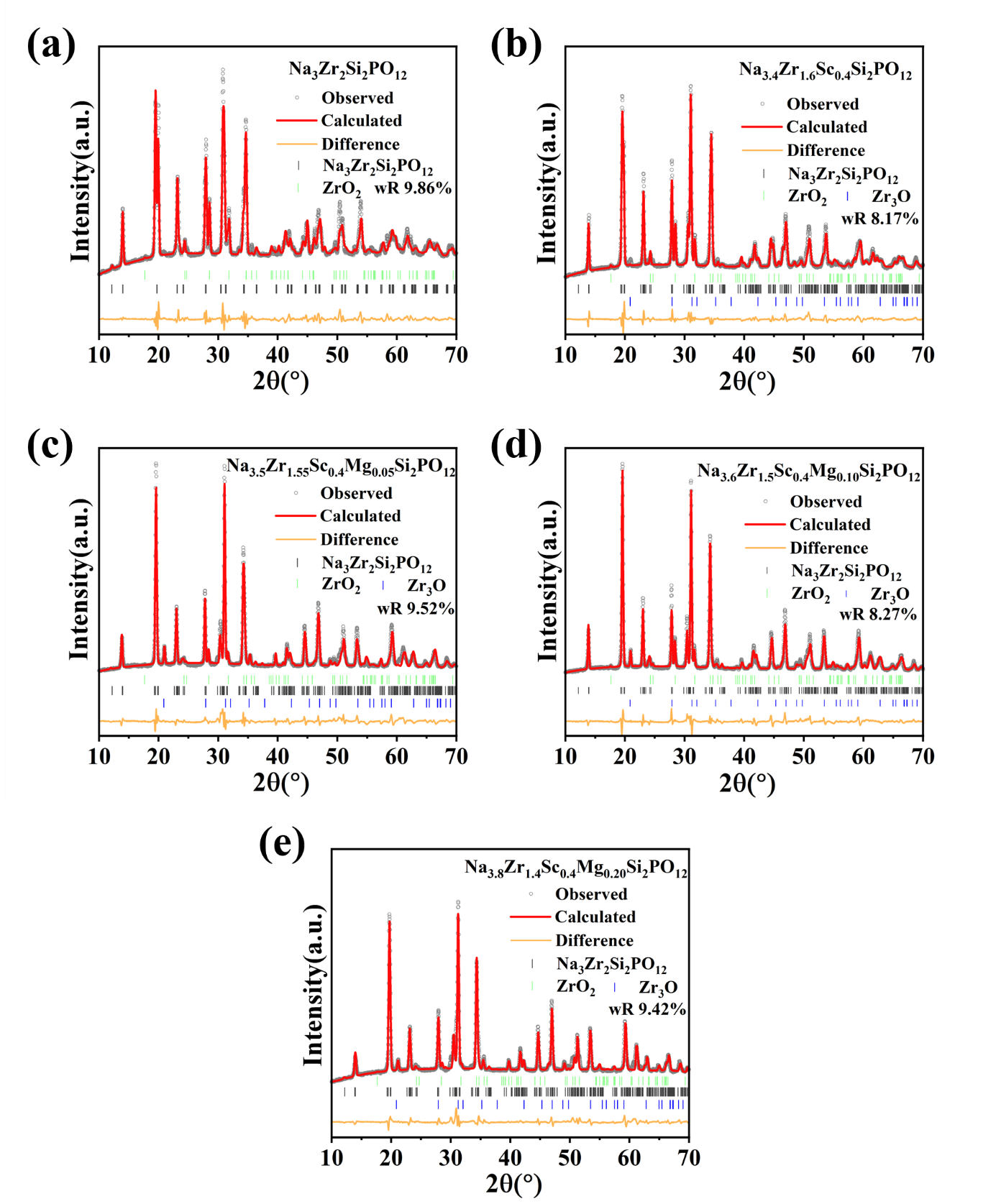


**Figure S2.** XRD Rietveld refinement of (a) Na_3_Zr_2_Si_2_PO_12_; (b) Na_3.4_Zr_1.6_Sc_0.4_Si_2_PO_12_; (c) Na_3.5_Zr_1.55_Sc_0.4_Mg_0.05_Si_2_PO_12_; (d) Na_3.6_Zr_1.5_Sc_0.4_Mg_0.10_Si_2_PO_12_; (e) Na_3.8_Zr_1.4_Sc_0.4_Mg_0.20_Si_2_PO_12_ samples.

**Table S1.** Unit cell lattice parameters for the all samples obtained from XRD refinement analysis.

| **Solid state electrolytes** | **a (Å)** | **b (Å)** | **c (Å)** | **β (°)** | **V (Å^3^)** | **wR** |
| --- | --- | --- | --- | --- | --- | --- |
| NZSP | 15.634 | 8.988 | 9.278 | 124.765 | 1071.084 | 9.86% |
| NSZSP | 15.636 | 9.048 | 9.142 | 124.517 | 1065.648 | 8.17% |
| NSZSP-0.05Mg | 15.668 | 9.108 | 9.099 | 124.858 | 1065.460 | 9.52% |
| NSZSP-0.10Mg | 15.707 | 9.100 | 9.125 | 124.995 | 1068.475 | 8.27% |
| NSZSP-0.15Mg | 15.741 | 9.120 | 9.120 | 125.233 | 1069.317 | 9.44% |
| NSZSP-0.20Mg | 15.726 | 9.115 | 9.115 | 125.153 | 1068.503 | 9.42% |


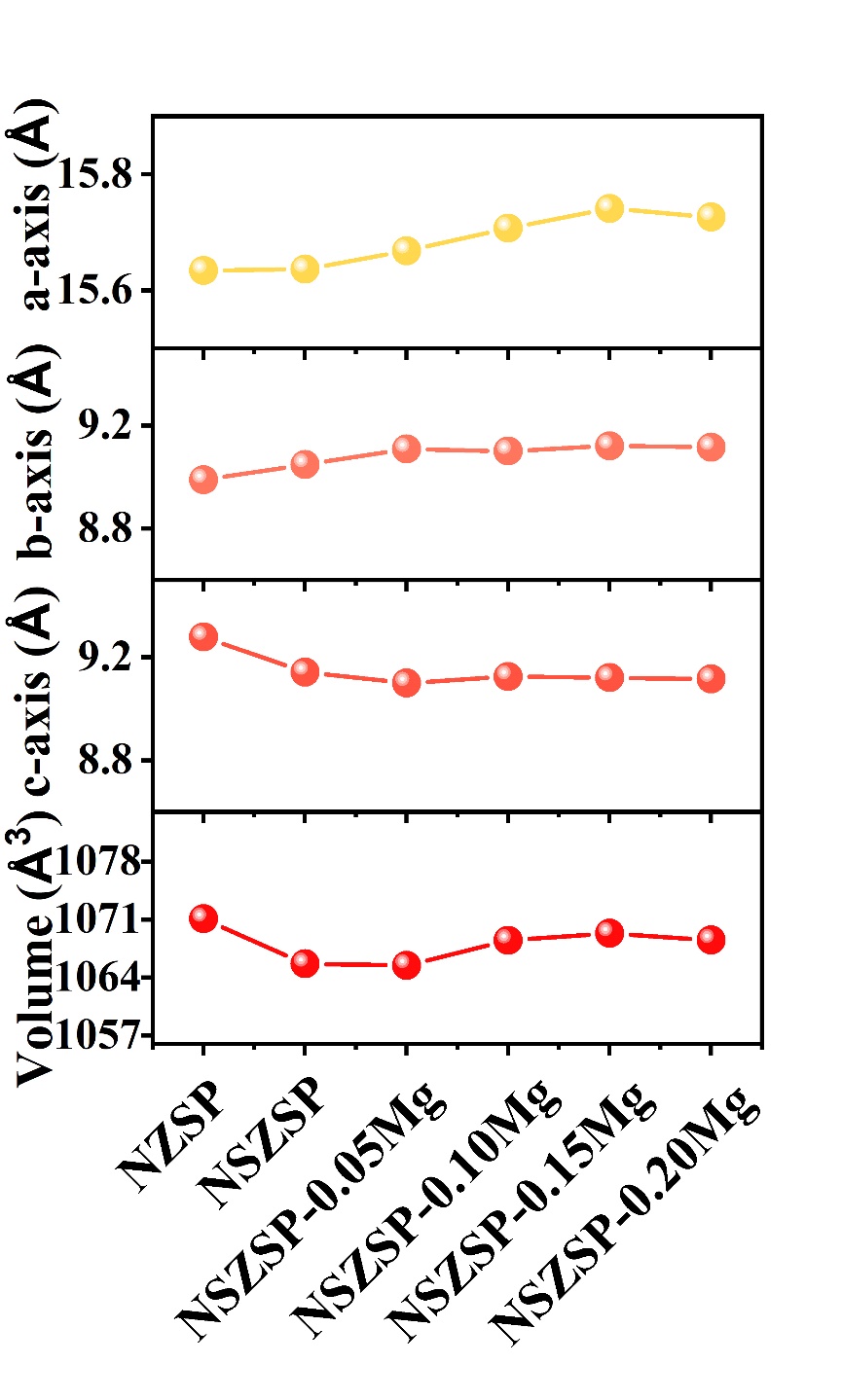


**Figure S3.** Variations in undoped and doped NZSP unit cell lattice parameters.


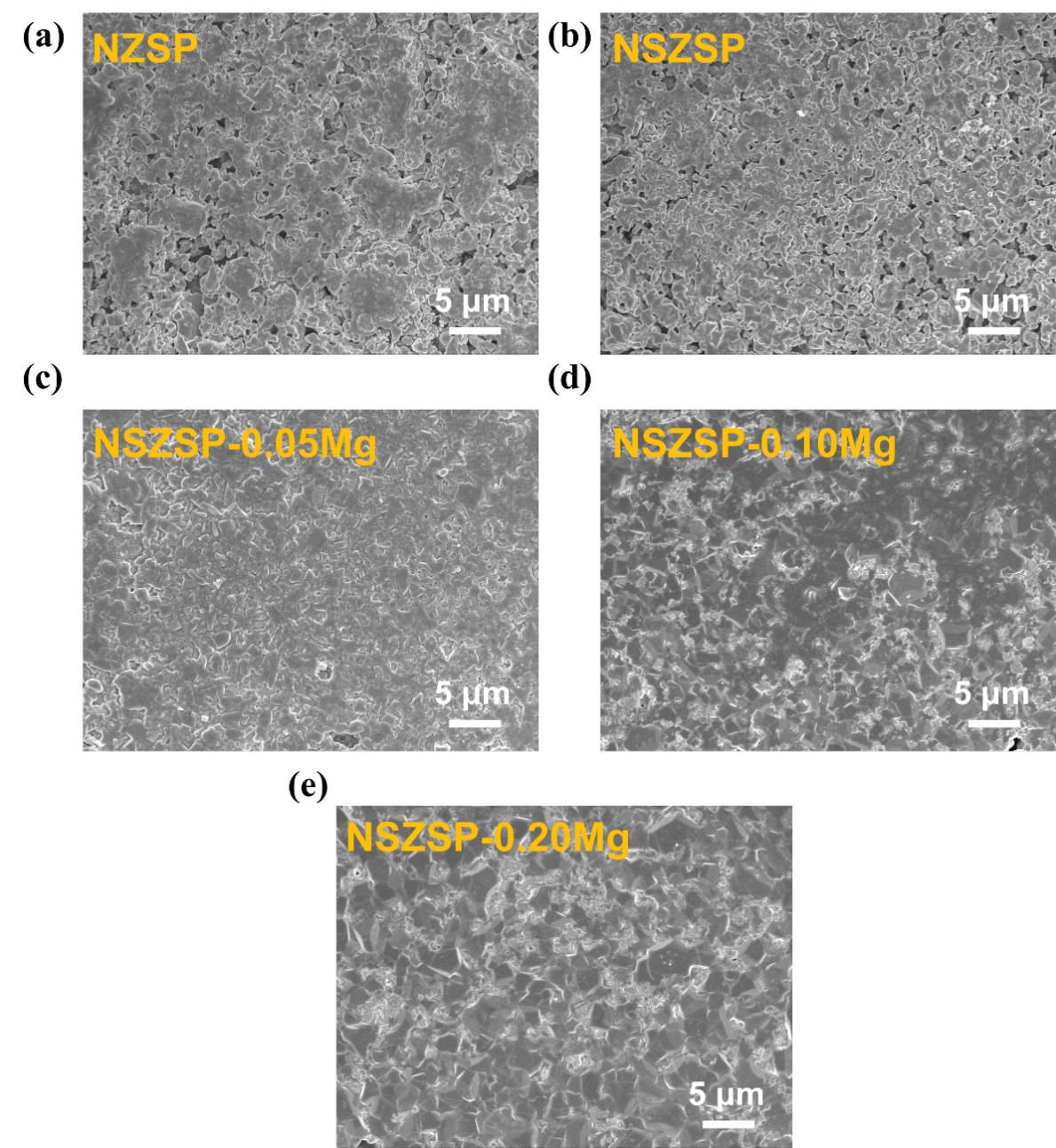


**Figure S4.** Top-view surface SEM images of (a) Na_3_Zr_2_Si_2_PO_12_; (b) Na_3.4_Zr_1.6_Sc_0.4_Si_2_PO_12_; (c) Na_3.5_Zr_1.55_Sc_0.4_Mg_0.05_Si_2_PO_12_; (d) Na_3.6_Zr_1.5_Sc_0.4_Mg_0.10_Si_2_PO_12_; (e) Na_3.8_Zr_1.4_Sc_0.4_Mg_0.20_Si_2_PO_12_ samples.


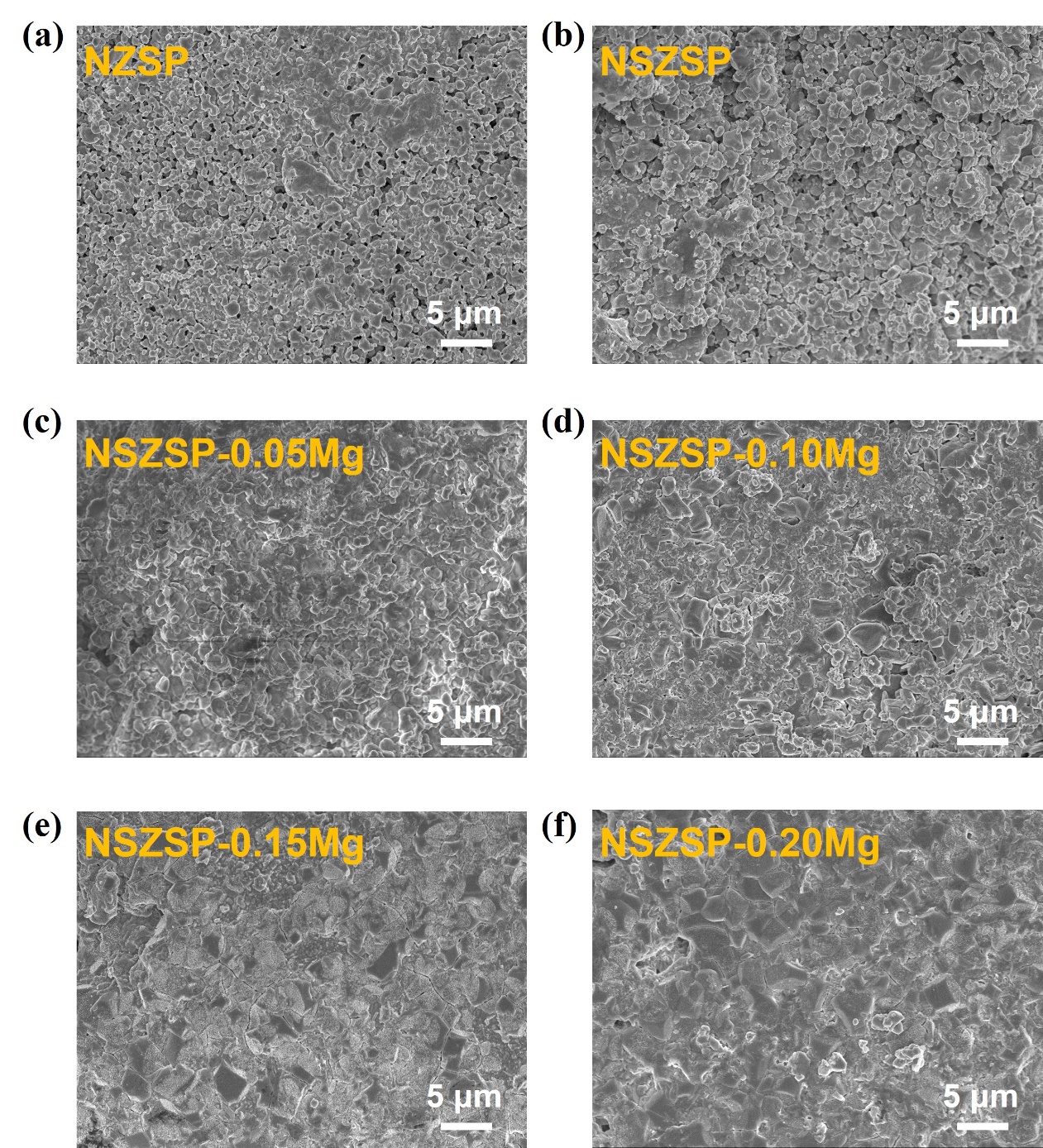


**Figure S5.** Cross-section view SEM images of (a) Na_3_Zr_2_Si_2_PO_12_; (b) Na_3.4_Zr_1.6_Sc_0.4_Si_2_PO_12_; (c) Na_3.5_Zr_1.55_Sc_0.4_Mg_0.05_Si_2_PO_12_; (d) Na_3.6_Zr_1.5_Sc_0.4_Mg_0.10_Si_2_PO_12_; (e) Na_3.7_Zr_1.45_Sc_0.4_Mg_0.15_Si_2_PO_12_ ;(f) Na_3.8_Zr_1.4_Sc_0.4_Mg_0.20_Si_2_PO_12_ samples.


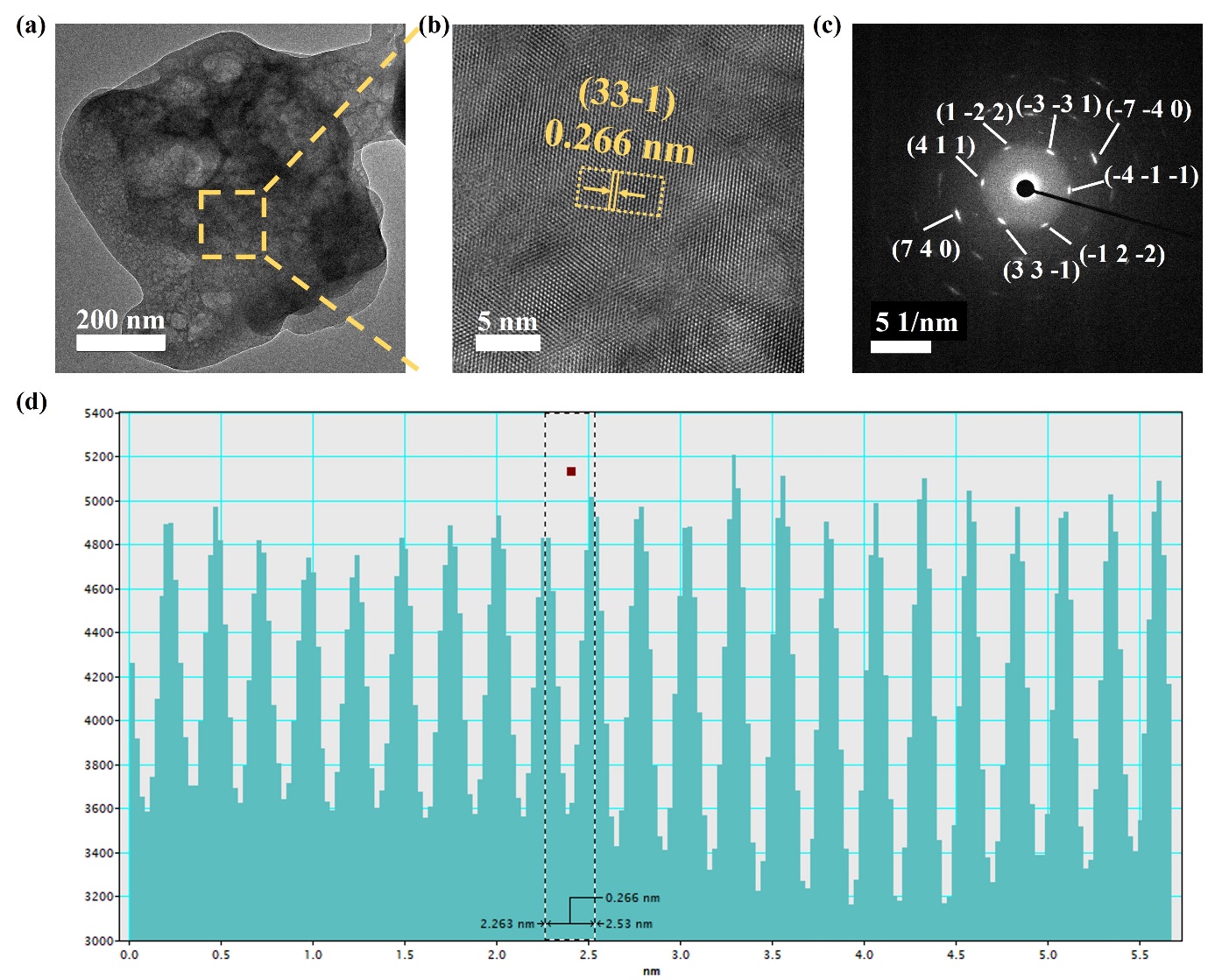


**Figure S6.** (a) TEM and (b) HRTEM of NSZSP-0.15Mg SSE. (c) SAED pattern. (d) The interplanar distances of (3 3 -1) planes.


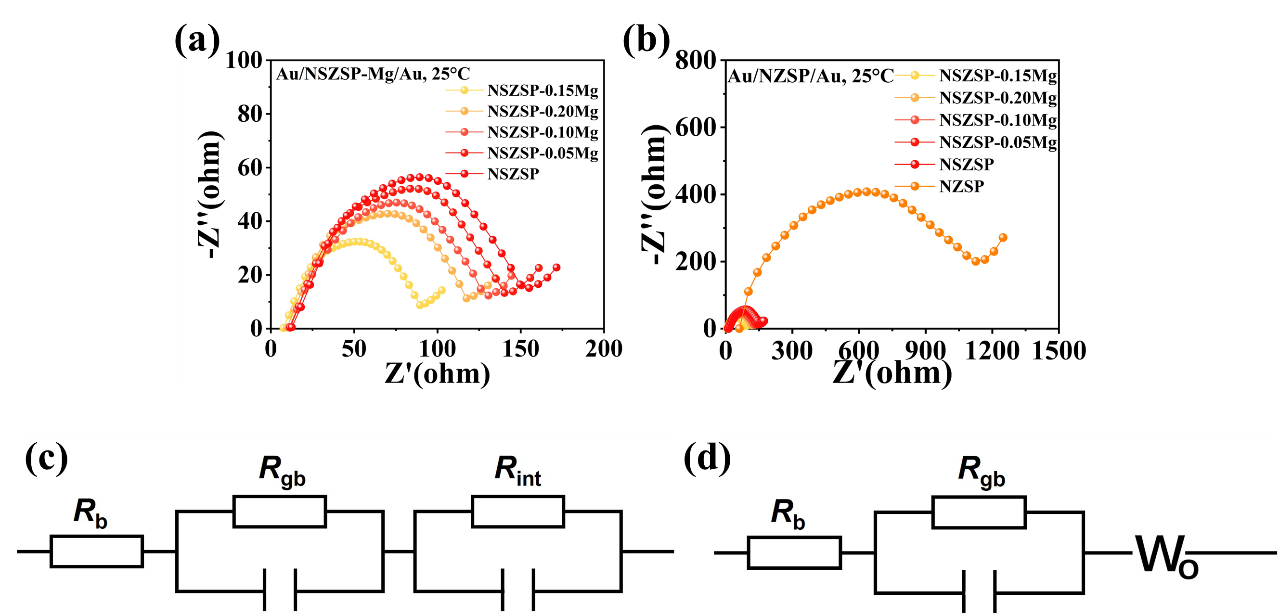


**Figure S7.** EIS measurements of the (a) NSZSP-xMg pellets and (b) NZSP pellet using Au film as blocking electrode for the determination of the conductivity. (c) and (d) Equivalent circuits for the battery cells.

**Table S2.** The resistances (R_b_, R_gb_, R_t_) and conductivities (σ_b_, σ_gb_, σ_t_) of the NSZSP-xMg and bare NZSP samples at 25℃.

| **Solid state electrolyte** | **NZSP** | **NSZSP** | **NSZSP-0.05Mg** | **NSZSP-0.10Mg** | **NSZSP-0.15Mg** | **NSZSP-0.20Mg** |
| --- | --- | --- | --- | --- | --- | --- |
| **Bulk grain resistance (R_b_)** | 61.2 | 12.6 | 11.6 | 11.1 | 7.5 | 9.8 |
| **Grain boundary resistance (R_gb_)** | 1064.6 | 142.5 | 128.7 | 119.5 | 82 | 107.7 |
| **Total resistance (R_t_)** | 1125.8 | 155.1 | 140.3 | 130.6 | 89.5 | 117.5 |
| **Bulk conductivity (σ_b_)** | 1.9×10^-3^ | 9.1×10^-3^ | 9.9×10^-3^ | 1.0×10^-2^ | 1.5×10^-2^ | 1.2×10^-2^ |
| **Grain boundary conductivity (σ_gb_)** | 1.1×10^-4^ | 8.0×10^-4^ | 8.9×10^-4^ | 9.6×10^-4^ | 1.4×10^-3^ | 1.1×10^-3^ |
| **Total conductivity (σ_t_)** | 1.0×10^-4^ | 7.4×10^-4^ | 8.2×10^-4^ | 8.8×10^-4^ | 1.3×10^-3^ | 9.8×10^-4^ |


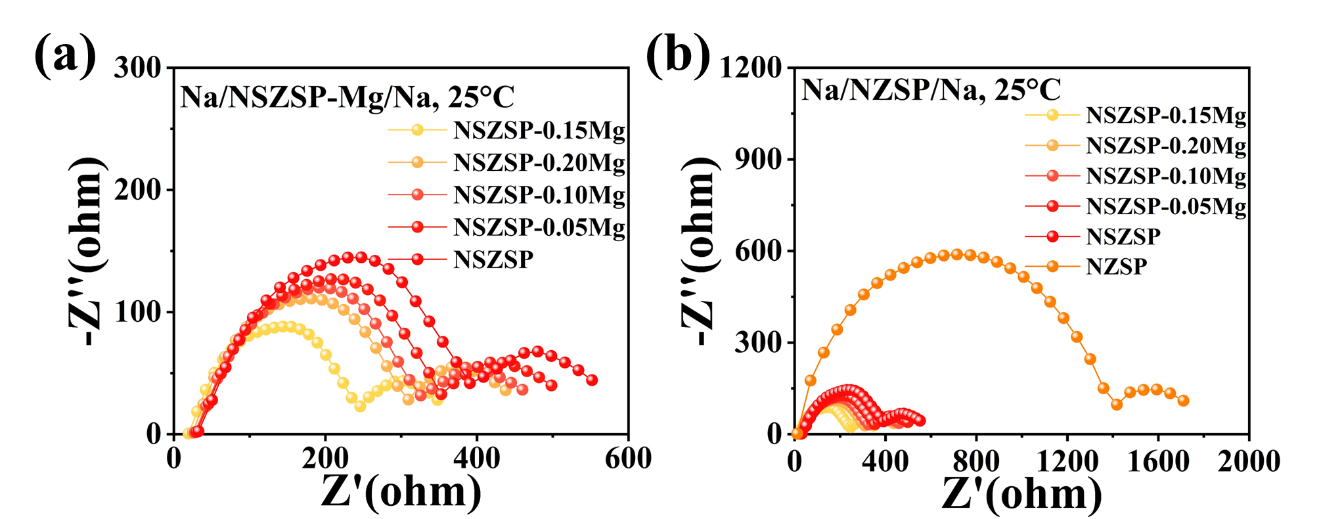


**Figure S8.** EIS measurements of the (a) NSZSP-Mg and (b) NZSP Na//NZSP//Na symmetric batteries at room temperature.

**Table S3.** The resistances (R_b_, R_gb_, R_int_, R_t_) of the Na//NZSP-xMg//Na and Na//NZSP//Na symmetric batteries at 25℃.

| **Solid state electrolyte** | **NZSP** | **NSZSP** | **NSZSP-0.05Mg** | **NSZSP-0.10Mg** | **NSZSP-0.15Mg** | **NSZSP-0.20Mg** |
| --- | --- | --- | --- | --- | --- | --- |
| **Bulk grain resistance (R_b_)** | 11.6 | 32.6 | 29.8 | 27.7 | 19.4 | 24.8 |
| **Grain boundary resistance (R_gb_)** | 1406.0 | 358.1 | 323.1 | 298.1 | 226.7 | 284.8 |
| **Interface resistance (R_int_)** | 292.9 | 161.2 | 145.4 | 134.2 | 102.0 | 128.2 |
| **Total resistance (σ_t_)** | 1710.5 | 551.9 | 498.3 | 460.0 | 348.1 | 437.8 |

**Table S4.** The Time-resolved resistances (R_b_, R_gb_, R_int_, R_t_) of the NSZSP-0.15Mg and bare NZSP samples at 25℃.

| **Solid state electrolyte** | **NSZSP-0.15Mg Fresh** | **NSZSP-0.15Mg 2^nd^ day** | **NSZSP-0.15Mg 4^th^ day** | **NSZSP-0.15Mg 6^th^ day** | **NSZSP-0.15Mg 8^th^ day** | **NSZSP-0.15Mg 20^th^ day** | **NZSP Fresh** | **NZSP**  **20^th^ day** |
| --- | --- | --- | --- | --- | --- | --- | --- | --- |
| **Bulk grain resistance (R_b_)** | 19 | 21 | 23 | 23 | 23 | 29 | 5 | 23 |
| **Grain boundary resistance (R_gb_)** | 246 | 270 | 295 | 300 | 302 | 369 | 567 | 2835 |
| **Interface resistance (R_int_)** | 102 | 112 | 122 | 124 | 126 | 153 | 117 | 586 |
| **Total resistance (σ_t_)** | 367 | 403 | 440 | 447 | 451 | 551 | 689 | 3444 |


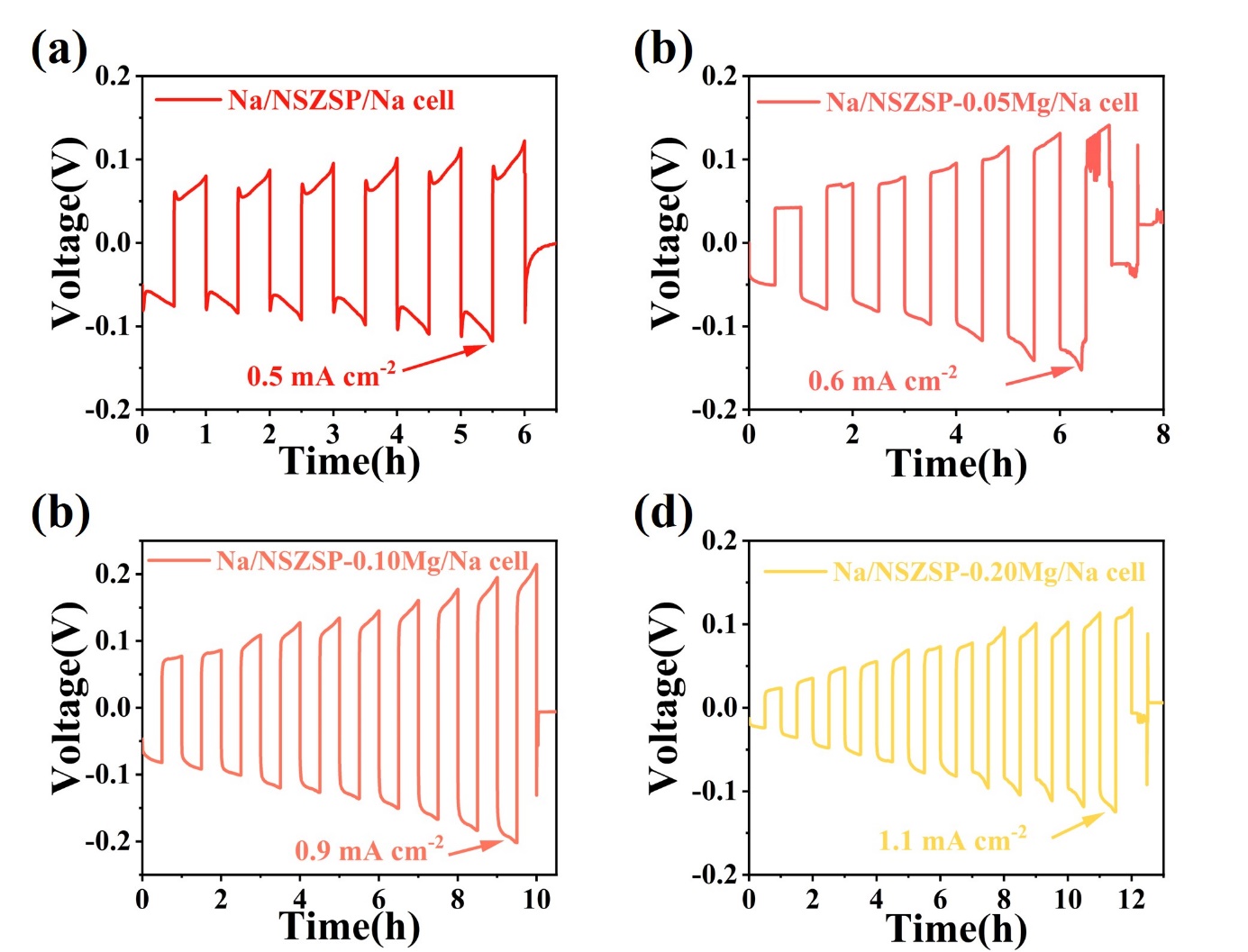


**Figure S9.** Critical current density test profile for the (a) NSZSP, (b) NSZSP-0.05Mg, (c) NSZSP-0.10Mg, (d) NSZSP-0.20Mg SSEs.


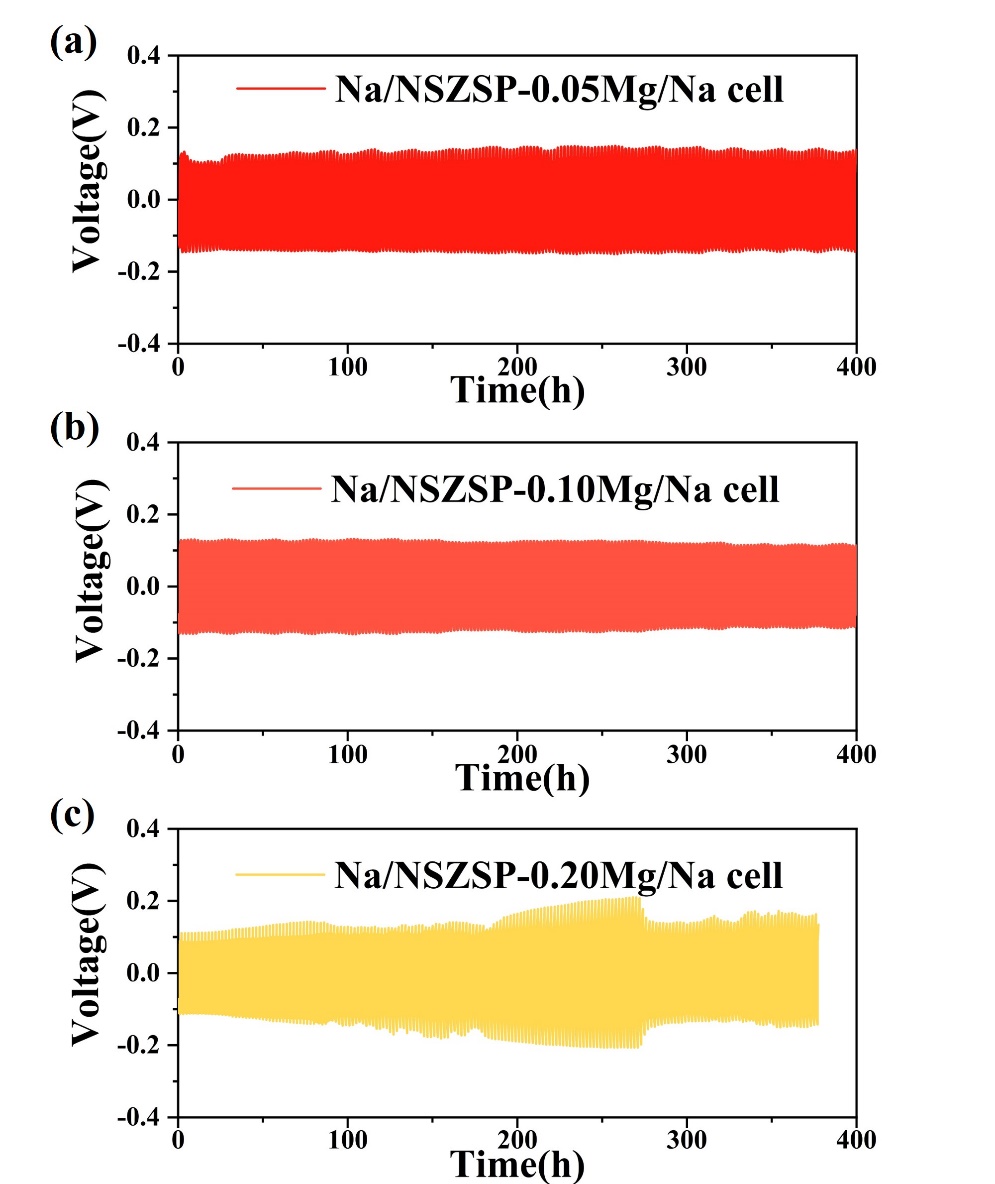


**Figure S10.** Galvanostatic cycling of Na//NZSP//Na symmetric battery with (a) NSZSP-0.05Mg, (b) NSZSP-0.10Mg and (c) NSZSP-0.20Mg SSEs at a current density of 0.5 mA cm^-2^.


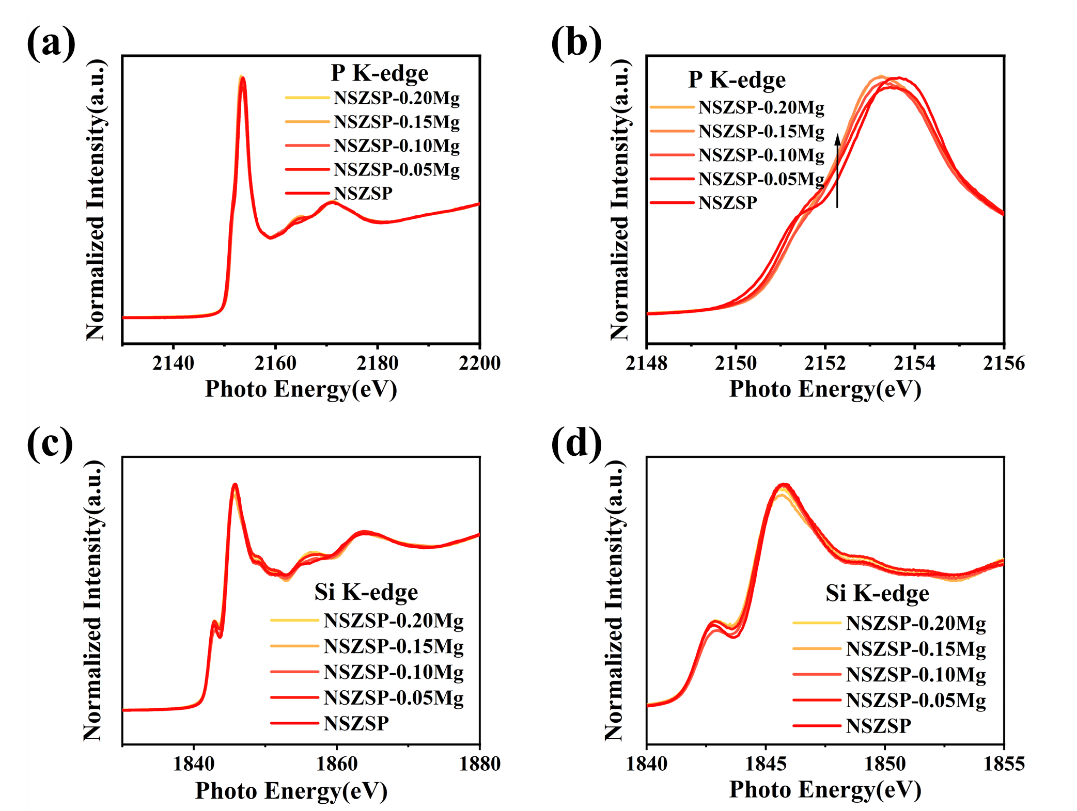


**Figure S11.** (a) P K-edge XANES spectra for NSZSP-Mg and (b) a magnified view of the selected energy region in P K-edge spectra. (c) Si K-edge XANES spectra for NSZSP-Mg and (d) a magnified view of the selected energy region in Si K-edge spectra.


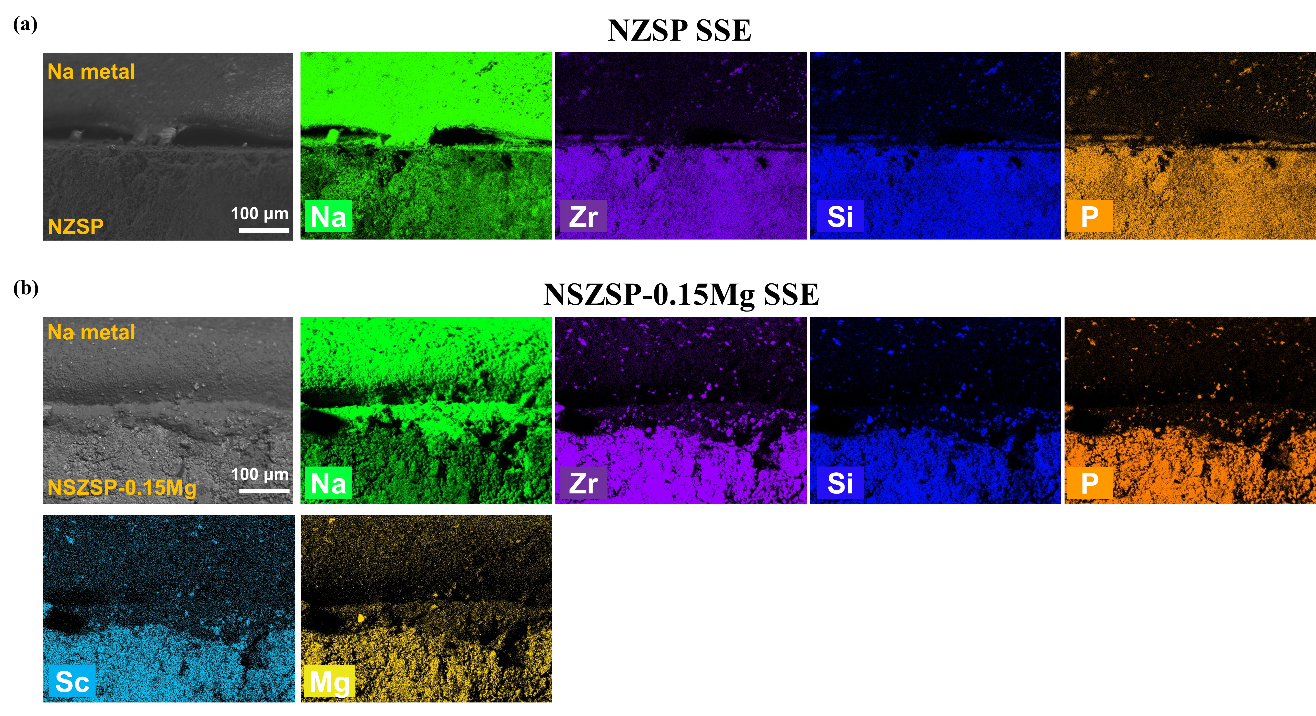


**Figure S12.** Cross-section elemental mapping images of the disassembled (a) Na//NZSP//Na cell and (b) Na//NSZSP-0.15Mg//Na cell after charge/discharge cycling.


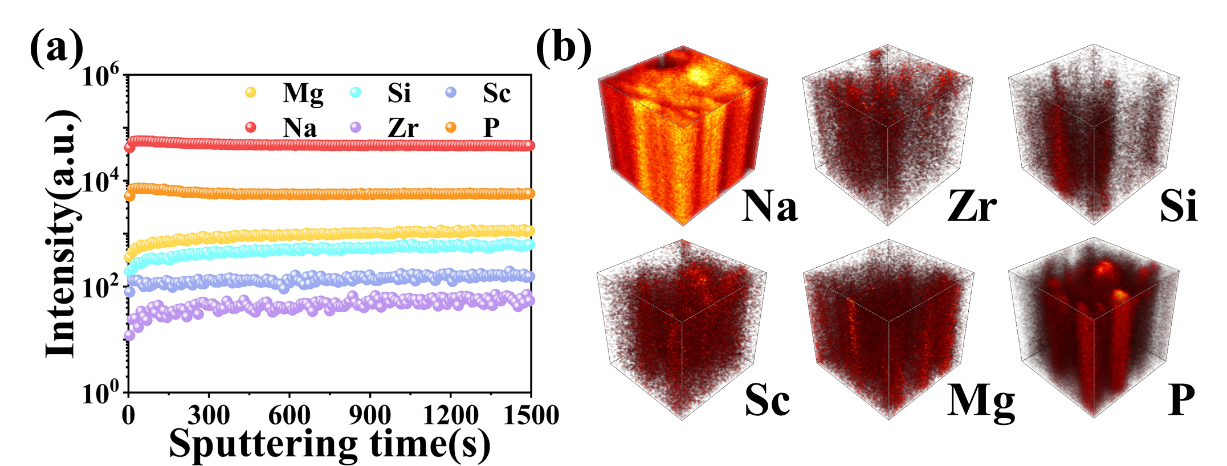


**Figure S13.** (a) In-depth ToF-SIMS profile of NSZSP-0.15Mg surface before Na plating/stripping cycles and (b) the corresponding 3D model images of Na, Zr, Si, Sc, Mg, P species.


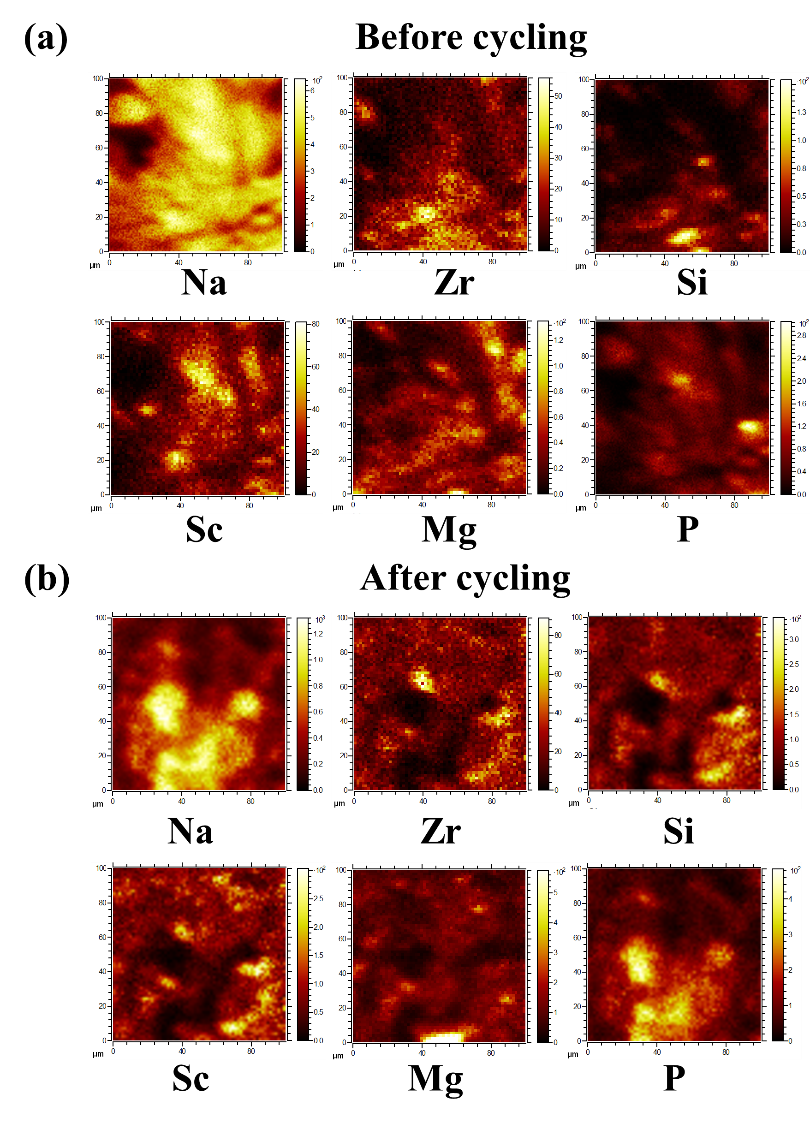


**Figure S14.** Integrated images of NSZSP-0.15Mg surface (a) before and (b) after Na plating/stripping cycles.


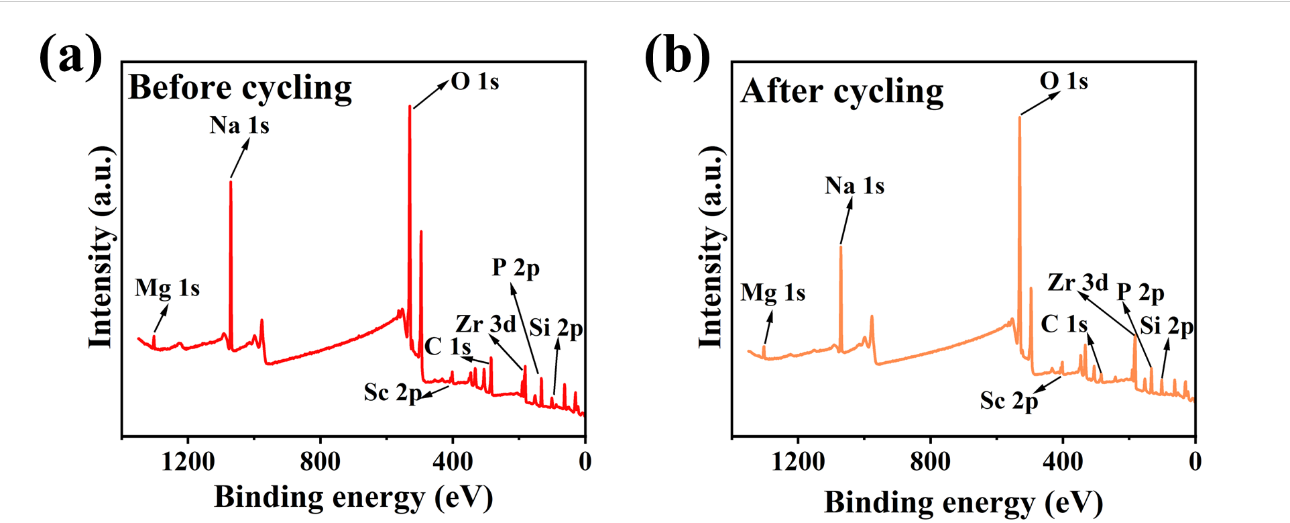


**Figure S15.** XPS survey spectra of (a) Before cycling and (b) After cycling.


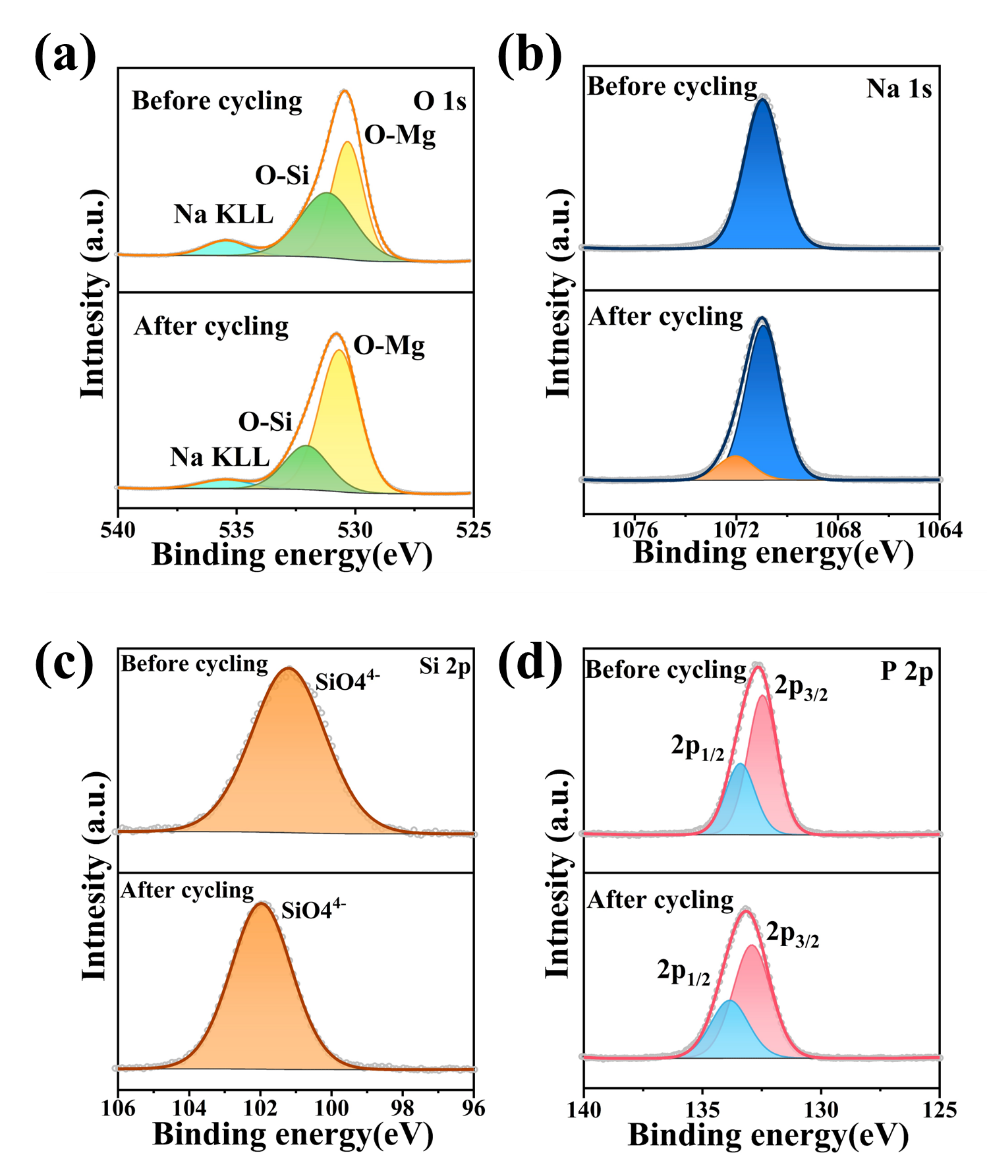


**Figure S16.** XPS spectra of NSZSP-0.15Mg before and after Na stripping/plating cycling: (a) O 1s; (b) Na 1s; (c) Si 2p; (d) P 2p.


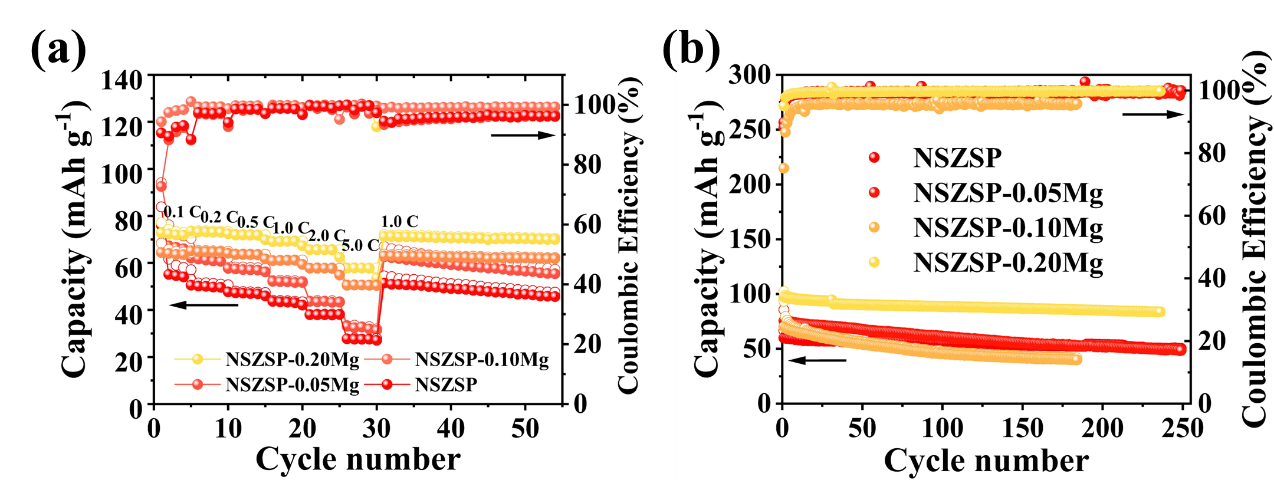


**Figure S17.** (a) Rate performance in the range of 0.1-5 C measured at 25℃. (b) Cycling performance comparison between NSZSP, NSZSP-0.05Mg, NSZSP-0.10Mg and NSZSP-0.20Mg SSEs.


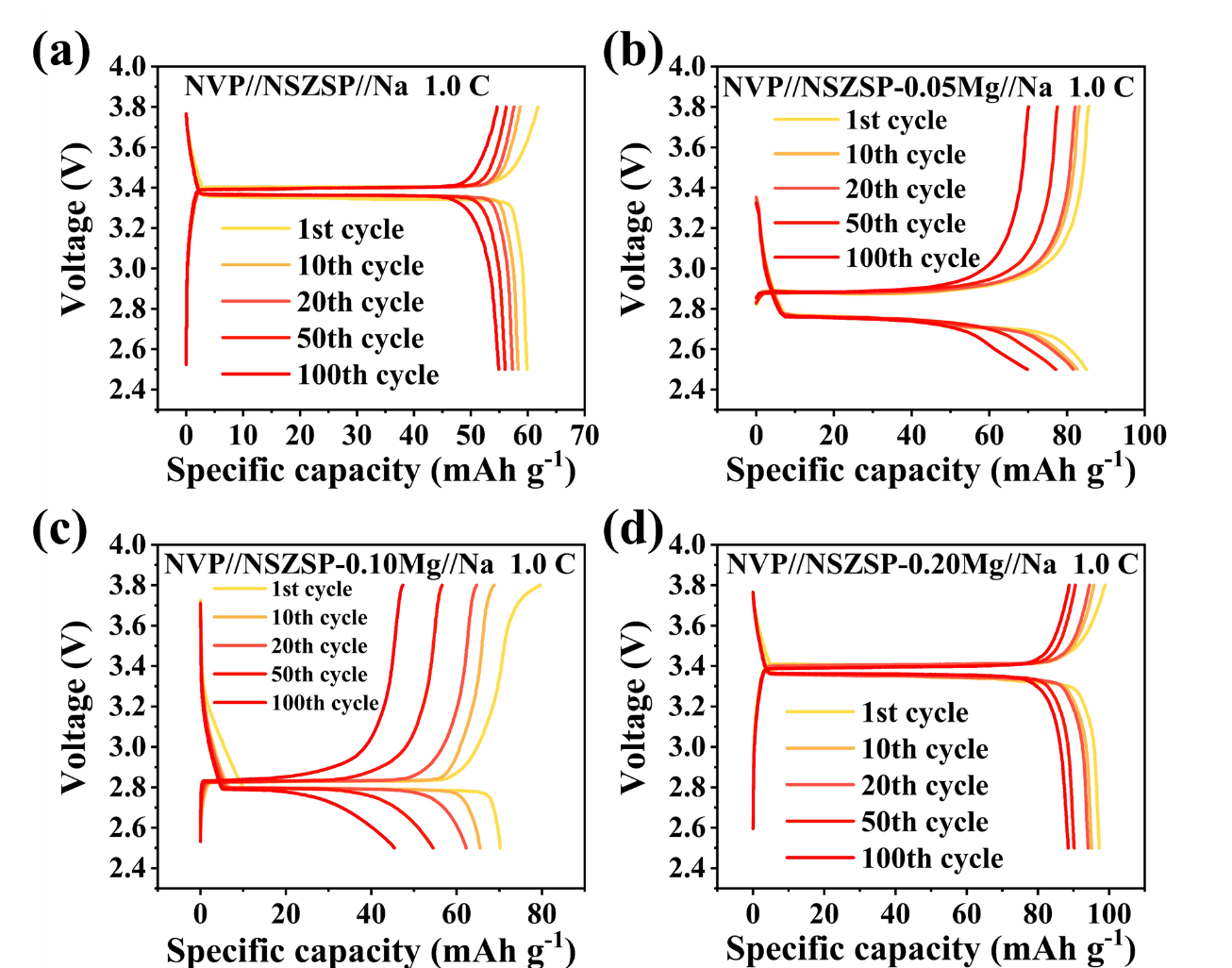


**Figure S18.** Galvanostatic charge/discharge cycling profile for the selected cycles of (a) NSZSP, (b) NSZSP-0.05Mg, (c) NSZSP-0.10Mg and (d) NSZSP-0.20Mg solid electrolytes at the rate of 0.1 C.

**Table S5** The resistances (R_SE_, R_ct_, R_cathode_, R_t_) of the NSZSp-0.15Mg and bare NZSP samples at 25℃ before and after rating.

| **Solid state electrolyte** | **NSZSP-0.15Mg**  **Before rating** | **NZSP**  **Before rating** | **NSZSP-0.15Mg**  **After Rating** | **NZSP**  **After Rating** |
| --- | --- | --- | --- | --- |
| **Bulk grain resistance (R_b_)** | 10 | 64 | 12 | 99 |
| **Grain boundary resistance (R_gb_)** | 209 | 533 | 756 | 1052 |
| **Interface resistance (R_int_)** | 896 | 2292 | 1500 | 2641 |
| **Total resistance (σ_t_)** | 1115 | 2889 | 2268 | 3792 |

**Table S6** Performance of SSMBs using Na_3_Zr_2_Si_2_PO_12_-based solid-state electrolytes.

| **Cell configuration** | **Doping element** | **Synthesis method** | **Cycling performance** | **Rate performance** | **Ref.** |
| --- | --- | --- | --- | --- | --- |
| Na_3_V_2_(PO_4_)_3_//Na_2.9_Zr_1.95_Mo_0.05_Si_2_PO_12_//Na | Mo | Solid-state reaction | 92.6 mAh g^-1^ at 100 mA g^-1^ after 1600 cycles | ~40 mAh g^-1^ at 500 mA g^-1^ | [1] |
| Na_3_V_2_(PO_4_)_3_//Na_2.7_Ca_0.15_Zr_2_Si_2_PO_12_//Na | Ca | Solid-state reaction | 96.8 mAh g^-1^ at 0.2C after 250 cycles | 88.95 mAh g^-1^ at 2C | [2] |
| Na_0.99_Zn_0.22_Fe_0.3_Mn_0.48_O_2_//Na_3.1_Zr_1.9_Sb_0.1_Si_2_PO_12_//Na | Sb | Sol-gel method | 95.96 mAh g^-1^ at 0.1C after 50 cycles | 72.3 mAh g^-1^ at 0.3C | [3] |
| Na_3_V_2_(PO_4_)_3_//Na_3.4_Zr_1.8_Mn_0.2_Si_2_PO_12_//Na | Mn | Solid-state reaction | 84.2 mAh g^-1^ at 0.1C after 260 cycles | 67.2 mAh g^-1^ at 1C | [4] |
| Na_3_V_2_(PO_4_)_3_//Na_3.65_Zr_1.675_Zn_0.2_Mg_0.125_Si_2_PO_12_//Na | Zn, Mg | Solid-state reaction | 76.56 mAh g^-1^ at 0.2C after 300 cycles | 32 mAh g^-1^ at 2C | [5] |
| Na_3_V_2_(PO_4_)_3_//Na_3.4_Zr_1.8_Ca_0.2_Si_2_PO_12_-G//Na | Ca | Solid-state reaction | 79.64 mAh g^-1^ at 1C after 500 cycles | 76.39 mAh g^-1^ at 1C | [6] |
| Na_0.67_Mn_0.47_Ni_0.33_Ti_0.2_O_2_//Na_3.12_Zr_1.94_Cu_0.06_Si_2_PO_12_//Na | Cu | Solid-state reaction | 75.8 mAh g^-1^ at 0.5C after 275 cycles | 52.7 mAh g^-1^ at 4C | [7] |
| Na_3_V_2_(PO_4_)_3_//Na_3.4_Zr_1.6_Sc_0.4_Si_2_PO_12_//Na | Sc | Solid-state reaction | 98.7 mAh g^-1^ at 1C after 300 cycles | 89.6 mAh g^-1^ at 1C | [8] |
| NaCrO_2_-PCE//Na_3.4_Zr_1.8_Cu_0.2_Si_2_PO_12_//Na | Cu | Solid-state reaction | 72.1 mAh g^-1^ at 1C after 660 cycles | 74.5 mAh g^-1^ at 10C | [9] |
| Na_3_V_1.5_Al_0.5_(PO_4_)_3_//Na_3.3_Zr_1.85_Ca_0.15_Si_2_PO_12_-G//Na | Ca | Solid-state reaction | 102.6 mAh g^-1^ at 0.5C after 275 cycles | 102 mAh g^-1^ at 0.5C | [10] |
| NaCrO_2_//Na_3.4_Zr_1.8_Mg_0.2_Si_2_PO_12_//Na | Mg | Solid-state reaction | 97.6 mAh g^-1^ at 1C after 1755 cycles | 102.6 mAh g^-1^ at 5C | [11] |
| Na_3_V_2_(PO_4_)_3_//Na_3.2_Zr_1.9_Ca_0.1_Si_2_PO_12_//Na | Ca | Sol-gel method | 94.9 mAh g^-1^ at 1C after 450 cycles | 80.5 mAh g^-1^ at 4C | [12] |
| Na_3_V_2_(PO_4_)_3_//Na_3_Zr_1.92_Ru_0.08_Si_2_PO_12_//Na | Ru | Solid-state reaction | 75.69 mAh g^-1^ at 0.3C after 100 cycles | 71 mAh g^-1^ at 0.5C | [13] |
| Na_3_V_2_(PO_4_)_3_//Na_3_Zr_1.9_Te_0.1_Si_2_PO_12_//Na | Te | Solid-state reaction | 105.49 mAh g^-1^ at 1C after 200 cycles | 104 mAh g^-1^ at 2C | [14] |
| Na_3_V_2_(PO_4_)_3_//Na_3.3_Zr_1.85_Zn_0.15_Si_2_PO_12_//Na | Zn | Solid-state reaction | 98.93 mAh g^-1^ at 0.5C after 200 cycles | 98.7 mAh g^-1^ at 2C | [15] |
| Na_3_V_2_(PO_4_)_3_//Na_3.1_Zr_2_La_0.1_Si_2_PO_12_//Na | La | Solid-state reaction | 101.3 mAh g^-1^ at 100 mA g^-1^ after 750 cycles | 117.3 mAh g^-1^ at 200 mA g^-1^ | [16] |
| Na_0.9_Zn_0.22_Fe_0.3_Mn_0.48_O_2_//Na_3.2_Zr_1.9_Sn_0.1_Si_2.2_P_0.8_O_12_//Na | Sn | Sol-gel method | 76.7 mAh g^-1^ at 0.1C after 300 cycles | 24.16 mAh g^-1^ at 1C | [17] |
| Na_3_V_2_(PO_4_)_3_//Na_3.7_Zr_1.45_Sc_0.4_Mg_0.15_Si_2_PO_12_//Na | Sc, Mg | Solid-state reaction | 75 mAh g^-1^ at 2C after 3000 cycles | 92.6 mAh g^-1^ at 5C | This work |

**Supplementary References**

[1] J. He, S. Yang, X. Xiao, D. Fang, R. Miao, C. Wang, L. Chen, N. Li, J. Li, Y. Su, H. Jin, *Energy Storage Materials* **2025**, 75, 104037.

[2] S. Guan, J. Lu, Y. Li, D. Xie, C. Zhuang, W. Zhang, *Ceramics International* **2025**, 51, 1172.

[3] M. Akbar, M. Kim, I. Moeez, A. H. Umar Bhatti, Y. H. Kim, J. Jeong, J.-Y. Kim, J.-H. Park, S. Yu, K. Y. Chung, *Chemical Engineering Journal* **2025**, 504, 158860.

[4] Y. Zhang, T. Gao, J. Yu, Y. Zhang, Y. Zhang, S. Chen, N. Xue, X. Zhang, Q. Yuan, L. Wang, W. Liu, *ACS Applied Materials & Interfaces* **2025**, 17, 10722.

[5] P. W. Jaschin, C. R. Tang, E. D. Wachsman, *Energy & Environmental Science* **2024**, 17, 727.

[6] J. Jia, T. Liu, Y. Li, J. Yang, H. He, X. Zhu, X. Yao, *ChemSusChem* **2024**, 17, e202400481.

[7] W. Wang, W. Yuan, Z. Zhao, D. Zou, P. Zhang, Z. Shi, J. Weng, P. Zhou, *Journal of Electroanalytical Chemistry* **2023**, 937, 117405.

[8] Q. Wang, C. Yu, L. Li, X. Liu, X. Zhang, G. Gao, Y. Wang, G. Li, *Energy Storage Materials* **2023**, 54, 135.

[9] D. Li, C. Sun, C. Wang, J. Li, Z. Wang, H. Jin, *Energy Storage Materials* **2023**, 54, 403.

[10] H. Jin, X. Xiao, L. Chen, Q. Ni, C. Sun, R. Miao, J. Li, Y. Su, C. Wang, *Advanced Science* **2023**, 10, 2302774.

[11] C. Wang, Z. Sun, Y. Zhao, B. Wang, C. Shao, C. Sun, Y. Zhao, J. Li, H. Jin, L. Qu, *Small* **2021**, 17, 2103819.

[12] Y. Lu, J. A. Alonso, Q. Yi, L. Lu, Z. L. Wang, C. Sun, *Advanced Energy Materials* **2019**, 9, 1901205.

[13] M. Dinachandra Singh, D. Kumar Gorai, K. Brajesh, P. Singh, V. Ranawade, A. Vijay Shinde, M. Jareer, R. Gupta, A. Garg, V. Agarwal, K. S. Nalwa, *Chemical Engineering Journal* **2024**, 489, 151330.

[14] L. Wang, S. Huo, Y. Lin, Y. Liu, Z. Wu, J. Song, C. Zhang, L. Liu, *Journal of Power Sources* **2024**, 624, 235604.

[15] L. Yang, H. Wang, Q. Liu, Z. Mei, L. Duan, H. Guo, *Journal of the European Ceramic Society* **2023**, 43, 4443.

[16] S. Yang, J. Liang, J. He, D. Fang, Y. Zhao, Y. Ding, A. Yin, Y. Li, C. Wang, J. Li, H. Jin, *ACS Applied Energy Materials* **2024**, 7, 9863.
